# Benchmarking cell-type-specific spatially variable gene detection methods

**DOI:** 10.1093/bib/bbag190

**Published:** 2026-04-27

**Authors:** Hui Yao, Shuai Mu, Fei He, Zhaoyuan Fang

**Affiliations:** Department of Colorectal Surgery and Oncology, the Second Affiliated Hospital, and Center for Biomedical Systems and Informatics, Zhejiang University-University of Edinburgh Institute (ZJU-UoE Institute), Zhejiang University School of Medicine, Zhejiang University, 866 Yuhangtang Road, Hangzhou 310000, Zhejiang, China; Senior Department of Oncology, The First Medical Center of Chinese People's Liberation Army (PLA) General Hospital, 28 Fuxing Road, Haidian District, Beijing 100039, China; Shanghai Neo-Biotechnology Co., Ltd., 518 XinZhuan Road, Shanghai 201612, China; Department of Colorectal Surgery and Oncology, the Second Affiliated Hospital, and Center for Biomedical Systems and Informatics, Zhejiang University-University of Edinburgh Institute (ZJU-UoE Institute), Zhejiang University School of Medicine, Zhejiang University, 866 Yuhangtang Road, Hangzhou 310000, Zhejiang, China; Edinburgh Medical School, College of Medicine and Veterinary Medicine, University of Edinburgh, Edinburgh BioQuarter, Little France Crescent, Edinburgh EH16 4TJ, United Kingdom

**Keywords:** spatial transcriptomics, cell-type-specific spatially variable genes, algorithm benchmarking

## Abstract

Cell-type-specific spatially variable genes (ctSVGs) integrate cell-type composition with spatially varying gene expression, revealing novel insights into molecular mechanisms through spatial transcriptomics. However, the emerging methods devoted to ctSVG detection have not been systematically evaluated, limiting their broader application and future algorithmic improvements. Here, we present a comprehensive evaluation of six state-of-the-art ctSVG detection methods, in terms of consistency, predictive performance, rotational robustness, scalability, and biological interpretability. We benchmark these methods on 46 real datasets, and a wide range of simulated datasets encompassing diverse biological and technical scenarios. Several key observations are yielded: (i) current algorithms complement each other in predictive performance and computational efficiency; (ii) STANCE and Celina achieve a better predictive performance on multiple spatial patterns, while C-SIDE, spVC, ctSVG, and CTSV exhibit tighter control of false positives; (iii) STANCE, ctSVG, CTSV, and Celina have better overall performance on single-cell resolution data; (iv) rotation invariance still warrants further investigation; (v) Celina appears to have a relative superiority in most metrics, though it tends to generate spurious signals affected by nontargeted cell types; and (vi) algorithmic choice strongly influences downstream biological interpretation. Together, our study offers valuable guidance for tool selection and algorithmic development.

## Introduction

Spatial transcriptomics, emerging as a revolutionary technology, effectively captures the spatial context of genomic expression heterogeneity hidden in bulk and dissociated single-cell sequencing approaches [1–7]. Notably, two related concepts of spatial gene expression variability warrant distinction because of their distinct biological interpretations. The first is spatially variable genes (SVGs), a general concept encompassing any heterogeneous spatial expression patterns observed in tissues, regardless of their sources of variation. Thus, a major limitation of SVGs is their intermingling with heterogeneous cell-type composition across spatial contexts, often biasing towards cell-type marker genes [8–10]. The second is cell-type-specific SVGs (ctSVGs), a more precise concept that focuses on spatial gene expression heterogeneity within the same cell type, thereby excluding the common bias originated from the frequently observed cell-type composition variability [11–13].

From the biological perspective, spatial transcriptomics offers a unique strength to identify ctSVGs, forming the basis of uncovering molecular modes constrained by tissue context. In cancer, malignant tissues show pronounced intra-tumor heterogeneity among both cancer and stromal/immune cells, driving cell-type-specific spatial variation in gene expression. For instance, in a recent study of small-cell lung cancer, ASCL1^+^ cancer subtype patients were associated with a poorer prognosis when containing ASCL1^+^NEUROD1^+^ regions with elevated expression of SLFN11 [14]. Prevalently, tumors occupy a core-edge architecture, where the epithelial core and invasive leading edges exert differential influences on transcriptional signatures of both cancer and surrounding cells. In head and neck squamous cell carcinoma, cancer cells with CLDN4 and SPRR1B overexpression tended to be at the core, and those with LAMC2 and ITGA5 overexpression at the edges [15, 16]. In kidney cancer, macrophages located at the tumor edges exhibited significantly higher IL1B expression than those in other spatial regions [17]. On the other hand, cancer cells and stromal/immune cells may have coincident or interactive patterns of spatial gene regulation. In colon cancer tissues, macrophages near REG1A^+^LCN2^+^ cancer cells and goblet cells were marked by SELENOP and STAB1 overexpression, whereas those near TGFBI^+^PERP^+^ cancer cells or FAP^+^ fibroblasts were marked by SPP1 [18, 19]. Additionally, heterogeneous neighborhoods could shape distinct gene expression signatures in cells, as exemplified by the cancer-associated fibroblasts [20]. The above modes of spatial expression are not restricted to malignant tissues. Similar regulatory schematics have also been widely observed in normal tissues [21–29], suggesting their widespread presence as common cellular adaptation principles across tissue states.

From the computational perspective, however, conventional algorithms were mainly devoted to SVGs rather than ctSVGs [13]. Among those SVG detection algorithms, there are kernel-based methods such as SpatialDE [30] (based on a Gaussian process) and SPARK [31] (based on a Poisson generalized linear spatial model), and graph-based methods such as scGCO (based on an undirected Delaunay-triangulation-transformed graph) [32] and Hotspot [33] (based on a directed K-nearest neighbor graph). Only recently, significant efforts have emerged to develop new computational approaches for identifying ctSVGs [11, 12, 21, 34–36]. C-SIDE [21] models spatial gene expression counts with a Poisson-lognormal distribution and, in its nonparametric mode, captures smooth spatial trends with thin plate spline basis functions. CTSV [11] employs a zero-inflated negative binomial distribution to model raw counts and combines three types of basis functions to approximate spatial patterns. spVC [34] also models spatial count data, while using generalized Poisson regression and quasi-Poisson estimation of overdispersion. spVC uses a two-step testing procedure: the first step identifies genes with a constant covariate effect and residual spatial effect, which are further tested for cell-type-specific spatial variability with a full model in the second step. Unlike C-SIDE and CTSV, spVC uses bivariate penalized spline basis functions over triangulation for spatial smoothing. In contrast, STANCE [36] and Celina [35] perform linear mixed-effect modeling on normalized expression data while using kernel functions to model spatial expression patterns. They also embrace two major differences. One is on how to handle random effects: STANCE decomposes spatial random effects by each cell type, whereas Celina partitions merely the cell-type-specific variance into spatial and nonspatial contributions. The other is on the use of kernels: STANCE leverages one distance-based Gaussian kernel, whereas Celina takes advantage of Gaussian, Matérn, and semi-parametric spline kernels to capture various types of spatial patterns. Another recent method, ctSVG [12], tailored for Visium HD and applicable to high-resolution single-cell-level spatial data, models normalized gene expression values with high computational efficiency by taking advantage of the PreTSA [37] regression framework with a shared design matrix across genes. Together, these approaches take the challenge of decomposing cell-type-specific spatial variances and provide powerful tools for spatial transcriptomic analysis.

Currently, a systematic evaluation of these ctSVG detection methods remains lacking, despite the many benchmarking studies for conventional SVG methods [38–42]. Thus, it is largely unclear how their performance varies with tissue compositions and spatial patterns, and what the computational efficiency and memory requirements are when scaled to large datasets. These issues highlight the necessity of a systematic comparative benchmarking beyond theoretical analysis.

To fill in the gap, we conducted a systematic evaluation of ctSVG detection methods under diverse experimental and simulation scenarios. We assessed their consistency and similarity, quantified their predictive performance, and evaluated their robustness to tissue section rotation. Furthermore, we benchmarked their computational efficiency in terms of runtime and memory consumption. Collectively, these analyses provide a practical guide for appropriate method choice in different scenarios and offer insights into the key issues underlying future methodological development.

## Materials and methods

### Lung cancer specimen preparation, sequencing, and data processing

Formalin-fixed paraffin-embedded (FFPE) tumor biopsies of six lung cancer patients (three LUAD and three LUSC) were obtained from the First Medical Center of Chinese People’s Liberation Army (PLA) General Hospital (see Table S1 for clinical information). All these samples were pretreated primary tumors. The LUAD and LUSC specimens were combined and mounted onto two 10$\times$ Genomics Visium HD slides, respectively. Hematoxylin and eosin (H&E) staining and imaging were performed following the Visium HD FFPE Tissue Preparation Handbook (CG000684). All procedures were performed according to the manufacturer’s instructions to preserve both tissue morphology and RNA integrity. Sample processing and library generation were performed following the Visium HD Spatial Gene Expression Reagent Kits according to the User Guide (CG000685). Briefly, the tissue sections were permeabilized to release mRNA molecules, which were hybridized to spatially barcoded probes on the two Visium HD slides. Reverse transcription, second-strand synthesis, and cDNA amplification were all performed on-slide. The sequencing libraries were then constructed, quantified, and finally sequenced on an Illumina sequencing platform according to the recommended paired-end sequencing configuration.

Raw sequencing data from above were first processed with the Space Ranger (3.0.1) for demultiplexing, alignment to the GRCh38-2020-A reference transcriptome, and generation of spatially resolved gene expression matrices with Visium Human Transcriptome Probe Set v2.0. Three bin sizes (2, 8, and 16 $\mu$m) were available from Space Ranger outputs for Visium HD data. All downstream analyses were performed on 2 $\mu$m resolution data. Cell binning was conducted using Space Ranger to obtain spatial gene expression profiles at single-cell resolution.

Since the computational resources required, such as those by CTSV and Celina, increase exponentially with the number of spots, we manually extracted a subset of spots for benchmarking purposes. Subsequently, we performed deconvolution using cell-type annotations and single-cell count matrices from the Human Lung Cell Atlas [43, 44] at https://cellxgene.cziscience.com/collections/6f6d381a-7701-4781-935c-db10d30de293.

### Metrics for method similarity

To systematically assess the similarity of results obtained from different ctSVG detection methods, we considered four complementary aspects: (i) the number of significant ctSVGs, (ii) concordance at the top (CAT) [45], (iii) global rank correlation, and (iv) overlap of significant ctSVGs. These metrics were selected to describe method similarity from multiple complementary perspectives, including detection sensitivity, agreement among top-ranked genes, global ranking consistency, and overlap of identified ctSVGs. As these metrics capture different methodological properties rather than defining performance superiority, they collectively facilitate a fair and comprehensive benchmarking.

The number of significant ctSVGs summarizes the overall detection sensitivity of each method under a fixed statistical threshold.

CAT was used to quantify concordance at the top of the ranked gene lists by measuring the proportion of shared genes among the top $k$ (from 1 to 200) ctSVGs ranked by two methods. Formally, for two methods $A$ and $B$, CAT$(k)$ is defined as


\begin{eqnarray*} & \mathrm{CAT}(k) = \frac{\left| T_{k}^{A} \cap T_{k}^{B} \right|}{k}, \end{eqnarray*}

where $T_{k}^{A}$ and $T_{k}^{B}$ denote the sets of the top $k$ ranked ctSVGs identified by methods $A$ and $B$, respectively. This metric emphasizes agreement among the most highly prioritized genes, which are typically of greatest biological relevance.

To capture global rank similarity, we computed Spearman’s rank correlation coefficients on gene-level *P*-value rankings. The coefficient $\rho$ was computed between the gene-wise rankings of *P*-values produced by two methods.

For gene list overlap, the Jaccard indices were computed upon several thresholds: significance levels (.05 and.01) or top-ranked genes (20 and 50). The Jaccard index for two ctSVG sets $A$ and $B$ is defined as


\begin{eqnarray*} & J(A,B) = \frac{\left| A \cap B \right|}{\left| A \cup B \right|}. \end{eqnarray*}

### Metrics for rotation invariance

To evaluate the robustness to tissue rotation, we quantified the consistency of ctSVG detection results before and after rotation using the same family of similarity measures.

CAT was used to assess the overlap of the top $k$ ctSVGs before and after rotation, capturing the stability of highly prioritized genes, Spearman’s correlation coefficient was used to evaluate the overall stability of *P*-value rankings, reflecting global preservations of gene ordering, and the Jaccard index was used to assess the overlap of ctSVG sets (either adjusted *P*-value $<.05$ or top 50 genes). Together, these metrics characterize robustness at various scales, ranging from top-ranked discoveries to genome-wide rankings, thereby providing a rigorous evaluation of rotation invariance.

### Metrics for predictive performance

Given the background truth, we further assessed predictive accuracy with the receiver operating characteristic (ROC) curve that plots the true positive rate (TPR) against the false positive rate (FPR), as well as the area under the curve (AUC) that summarizes classification performance irrespective of significance thresholds. Formally, the TPR and FPR are defined as


\begin{eqnarray*} & \mathrm{TPR} = \frac{\mathrm{TP}}{\mathrm{TP} + \mathrm{FN}}, \qquad \mathrm{FPR} = \frac{\mathrm{FP}}{\mathrm{FP} + \mathrm{TN}}, \end{eqnarray*}

where TP, FP, TN, and FN denote the numbers of true positives, false positives, true negatives, and false negatives, respectively.

Higher AUC values indicate a stronger capability to distinguish true ctSVGs from non-ctSVGs, which is particularly useful under class imbalance [46]. The AUC corresponds to the probability that a randomly chosen true ctSVG is ranked higher than a randomly chosen non-ctSVG, providing a threshold-independent summary of classification performance.

Similarly, we also reported sensitivity, defined as the proportion of true positives correctly identified. Sensitivity is equivalent to TPR and is given by


\begin{eqnarray*} & \mathrm{Sensitivity} = \frac{\mathrm{TP}}{\mathrm{TP} + \mathrm{FN}}. \end{eqnarray*}

To evaluate false positive control, we conducted a null model analysis by randomly permuting spatial coordinates 100 times on the SeqFish+_cortex dataset, thereby neutralizing any potential spatial structures. Under this null model, the empirical FPR was computed as


\begin{eqnarray*} & \mathrm{FPR}_{\mathrm{null}} = \frac{\mathrm{Number of ctSVGs detected under permutation}}{\mathrm{Total number of genes tested}}. \end{eqnarray*}

The proportion of falsely detected ctSVGs under this permutation served as the empirical FPR, and specificity was naturally evaluated as $1 - \mathrm{FPR}$. That is,


\begin{eqnarray*} & \mathrm{Specificity} = 1 - \mathrm{FPR}. \end{eqnarray*}

### Metrics for scalability

To assess the computational performance of each method in terms of CPU usage and memory consumption, we conducted experiments on a high-performance computing node equipped with dual AMD EPYC 9654 96-Core Processors (Genoa architecture, base frequency 2.40 GHz). Before execution, we loaded the RhpcBLASctl package in R and set the number of BLAS threads to 1 using blas_set_num_threads(1), while we restricted the computational resources to a *single CPU core* for all method executions. The runtime and peak memory usage were monitored with system.time() and gc() functions, respectively.

## Results

### Overview of cell-type-specific spatially variable gene methods

C-SIDE [21] models gene expression in each spot as a weighted sum of expected cell-type-specific signatures, where weights correspond to the estimated cell-type proportions. These cell-type-specific signatures are represented as a linear combination of $L$ covariates, which are provided by the user via a design matrix to explain various types of differential expression: with categorical and continuous variables, it could detect expression changes between spatial regions or affected by the density of other cell types; and with smooth basis functions, it could identify ctSVGs nonparametrically, where a larger number of smooth basis functions corresponds to a higher effective degrees of freedom for finer spatial resolution.

CTSV [11] models the raw counts in each spot using a zero-inflated negative binomial distribution to account for over-dispersion and excessive zeros, where the logarithmic mean parameter is expressed as a combination of cell-type-specific spatial effects (respectively, on the 2D) weighted by estimated cell-type proportions. CTSV employs five basis functions from three types to capture spatial variation, and performs Wald tests on the coefficients of these basis functions. The five *P*-values are subsequently combined with the Cauchy combination rule.

spVC [34] constructs a Delaunay triangulation mesh to approximate the irregular spatial layout of spots. In the corresponding TriMesh function, a critical parameter controlling the resolution of the mesh, $n$, was set to 2 as recommended by the tutorial. On this triangulation mesh, spatial basis functions are then defined over each triangle to represent smooth variation in local space. Subsequently, spVC basically fits a generalized additive model with spatially varying coefficients and estimates the model parameters using a joint quasi-likelihood approach. spVC has a two-step design by default: prefiltering with a simplified model for an overall spatial effect, and then testing candidate genes using the full model for specific spatial effects. In practice, spVC supports both the two-step strategy (prefiltering and fitting only candidate genes on the full model) and the one-step strategy (no prefiltering and fitting all genes on the full model). In this study, we compare both strategies. The two-step and one-step versions are denoted as spVC_2 and spVC_1, respectively.

STANCE [36] employs a variance component framework to decompose gene expression variation into nonspatial components (fixed effects) and multiple cell-type-specific spatial components (random effects). The spatial effects, modeled by Gaussian kernel matrices based on Euclidean distance, are rotation-invariant by design. The detection procedure also proceeds in two stages: first, identifying both SVGs and ctSVGs, and then subsequently dissecting into ctSVGs.

Celina [35] takes advantage of a spatially varying coefficient framework, where the effect of spatial location on expression is captured using multiple spatial kernels, including one spline kernel, five Gaussian kernels, and five Matérn kernels. Effects attributed to the proportions of nontarget cell types are accounted for by fixed effects in the linear mixed model. For a target cell type, a null model is fitted for each kernel, using restricted maximum-likelihood estimation, with parameters iteratively updated via the average information algorithm. Finally, Celina integrates $P$-values across multiple kernels to identify ctSVGs specific to the target cell type.

ctSVG [12] models normalized single-cell-level gene expression values and adopts the PreTSA [37] regression framework with a shared design matrix across genes to achieve high computational efficiency, which is preferred for large-scale high-resolution data. PreTSA represents spatial patterns as smooth functions of spatial coordinates spanned by multiple cubic B-spline basis functions, and estimates regression coefficients via least squares. For each gene in a given cell type, spatial variability is assessed using F statistics to test whether any of the spline coefficients significantly deviates from zero.

While C-SIDE [21], Celina [35], STANCE [36], CTSV [11], and spVC [34] are all applicable to spatial transcriptomics data at both spot and single-cell level, ctSVG [12] was specifically developed for Visium HD data and only supports analyses at single-cell resolution.

For spot-level data, cell-type proportions are required as input, whereas for single-cell level data, cell-type labels are needed. In this study, all datasets were deconvoluted with RCTD [22], a popular deconvolution tool with favorable performance in previous evaluations [47–49].

### Benchmarking framework

Six state-of-the-art methods were included in this benchmarking study: C-SIDE [21], Celina [35], STANCE [36], spVC [34], CTSV [11], and ctSVG [12]. These methods use cell-type proportions and spatial transcriptomic data as input and model the spatial expression variation of individual genes. They either work on raw counts or normalized data (Table 1). Based on their spatial smoothing strategies, these methods can be broadly categorized into two groups: basis-function-based and kernel-based. These methods also differ in the resolutions of input data: ctSVG accepts only single-cell resolution, whereas the other methods support both spot-level and single-cell-level data.

**Table 1:** Comparison of six ctSVG computational methods

Method	Publication	Spatial smoothing	Data	Resolution	Hypothesis testing
C-SIDE	Nature Methods, 2022 [21]	Basis functions	Raw counts	SP, SC^a^	Wald test
CTSV	Bioinformatics, 2022 [11]	Basis functions	Raw counts	SP, SC	Wald test
spVC	Genome Biology, 2024 [34]	Basis functions	Raw counts	SP, SC	LRT + Wald test^b^
Celina	Nature Communications, 2025 [35]	Kernel functions	Normalized	SP, SC	Scaled Chi-square
STANCE	Nature Communications, 2025 [36]	Kernel functions	Normalized	SP, SC	Scaled Chi-square
ctSVG	Genome Biology, 2025 [12]	Basis functions	Normalized	SC	F test

^a^SP: spot bulk resolution; SC: single-cell resolution. ^b^Fixed effect: Likelihood ratio test (LRT); Random effect: Wald test.

We compiled 40 public real datasets from seven platforms (Fig. S1, See Supplementary Information for website links and references), including Microarray-based Spatial Transcriptomics (ST) [50], Visium (10$\times$) [51, 52], MERFISH [53–55], Slide-seq [56], Slide-seqV2 [57], Stereo-seq [58], and seqFISH+ [59]. These datasets span a wide range of resolutions and include both imaging- and sequencing-based technologies. The paired single-cell reference datasets were also collected for use in deconvolution. Additionally, we performed Visium HD (10$\times$) sequencing with specimens from six lung cancer patients (Table S1), including three lung adenocarcinomas (LUAD) and three squamous cell carcinomas (LUSC). Some platforms, such as Stereo-seq, have very high resolution, generating tens of thousands of spatial spots, which can exceed the computational capacity of certain methods. To ensure fair benchmarking, slices were cropped and subsetted so that all methods could complete within a reasonable time (See Supplementary Information for details).

**Figure 1:**
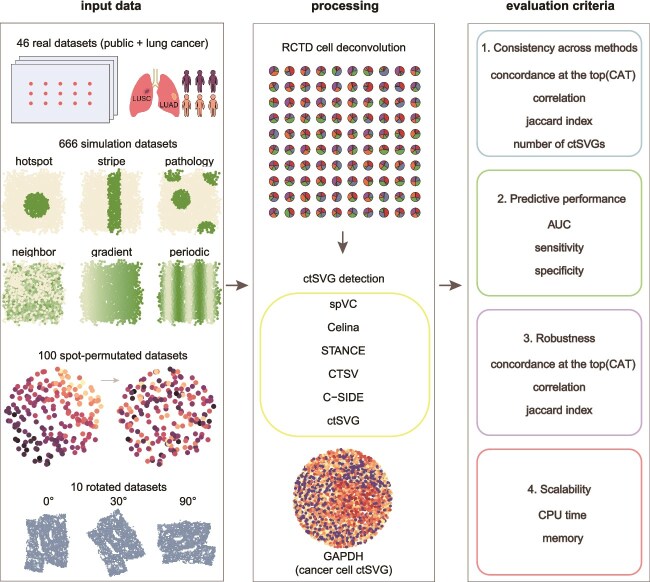
Evaluation framework of ctSVG detection methods. Datasets for this study include 46 real datasets, 666 simulated datasets, 100 spot-permutated datasets, and 10 coordinate-rotated datasets. All spot level and partial single-cell level datasets were deconvoluted with RCTD, followed by ctSVG analysis. Six state-of-the-art methods (spVC, Celina, STANCE, CTSV, C-SIDE, and ctSVG) were evaluated in terms of the following key aspects: (1) cross-method consistency; (2) predictive performance; (3) robustness to slice rotation; (4) scalability in terms of runtime and memory. In this flowchart, GAPDH is an example tumor-cell-specific ctSVG in melanomas (tissue section MBM13) [62].

Since real datasets typically lack ground truth, we further generated synthetic datasets for objective evaluation of algorithmic performance, including 666 simulated datasets, 10 coordinate-rotated datasets, as well as 100 randomized null-model datasets (Fig. 1, see Supplementary Information for details).

We conducted a systematic analysis on all these datasets with each method, summarized their results, and evaluated their performance in terms of the following criteria (Fig. 1): (i) consistency across methods; (ii) predictive performance on background truth; (iii) robustness to slice rotation; and (iv) scalability.

### Consistency of cell-type-specific spatially variable gene detection on real datasets

To evaluate the consistency of ctSVG methods, we compared the ctSVGs identified across 36 real datasets of either spot or single-cell resolution (summarized in Table S2). The comparison was conducted in terms of the number of identified genes, overlap proportion, Jaccard index, correlation, and CAT [45]. For methods using a two-step test, we assigned a *P*-value of 1 to genes filtered out in the first step. The one-step and two-step versions of spVC are denoted as spVC_1 and spVC_2, respectively, and these notations are used throughout the paper. In this comparison, spVC_1 was used as spVC_2 failed to identify significant genes, particularly in data with single-cell resolution.

**Figure 2:**
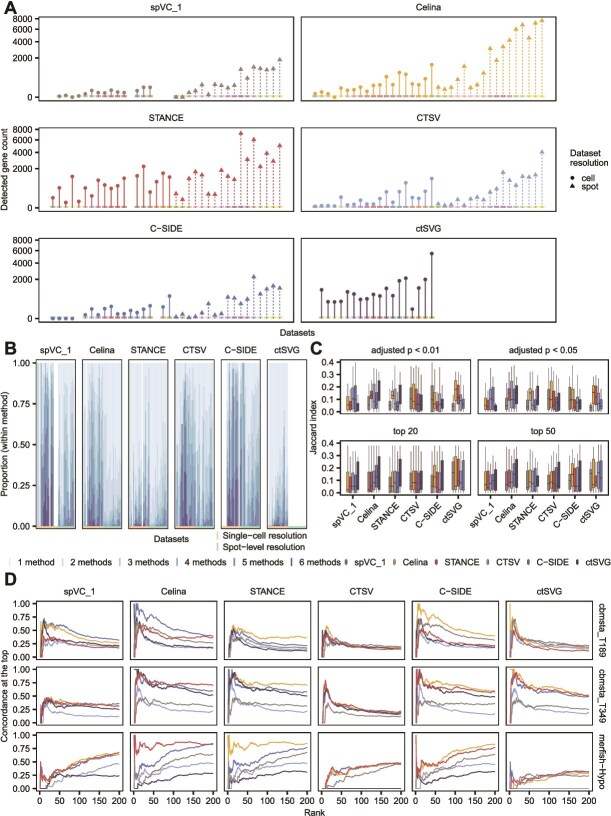
Benchmarking on real datasets. (A) Number of ctSVGs detected by each method (adjusted $P <.05$). The intensity-coded bar along the x-axis identifies the dataset. Circular point: single-cell resolution data; triangular points: spot-level resolution data. The one-step and two-step versions of spVC are denoted as spVC_1 and spVC_2, respectively. Since spVC_2 failed to identify significant genes in data with single-cell resolution, only spVC_1 has been included in the comparison in this section. (B) Proportion of significant genes covered by other methods in different degrees (from 1 to 6 methods, shading intensity from light to strong, respectively). Each small bin in each method represents one dataset in the same order as panel (A) . (C) Pairwise Jaccard index of ctSVGs identified at different significance levels (.05 and.01) or ranking thresholds (top 20 and 50). (D) Pairwise CAT (top 200 genes ranked by adjusted *P*-values) for the three datasets shown in the figure.

As shown in Fig. 2A, at a significance level of 0.05 on adjusted *P*-values, the number of ctSVGs identified varied considerably across methods. For example, spVC_1 reported the smallest number of ctSVGs and identified almost no ctSVGs in multiple datasets. On the other hand, STANCE, Celina, and ctSVG (single-cell resolution only) reported a greater number of ctSVGs. Furthermore, for each method, we classified its ctSVGs into six groups: those unique in themselves and those shared by 2–6 methods (Fig. 2B). C-SIDE, CTSV, and spVC_1 exhibited a higher proportion of ctSVGs in agreement with other methods, reflecting more conservative results, whereas most ctSVGs identified by ctSVG, STANCE, and Celina are method-specific. It is worth noting that, as the background truth is not available for real datasets, the differences among methods only reflect their similarity and uniqueness, rather than accuracy or superiority.

Beyond ctSVG counts and proportions, we further evaluated the methods using multiple similarity metrics. We first calculated the Jaccard indices at different significance thresholds (0.05 and 0.01) and for the top-ranked genes (20 and 50) (Fig. 2C). Although Jaccard indices between methods were generally low (often below 0.2), Celina, ctSVG, and STANCE appeared to have stronger similarity in terms of significant ctSVGs, and Celina and C-SIDE also exhibited higher consistency in terms of top ctSVGs. We then calculated correlation coefficients using the ranks of all genes. Overall, STANCE, Celina, and ctSVG exhibited high correlation, while high pairwise concordance was also evident in individual datasets (Figs S2–S5). Several pairs of methods (STANCE and CTSV, STANCE and Celina, C-SIDE and CTSV, C-SIDE and spVC_1, or Celina and spVC_1) showed good consistency on certain datasets. In addition, higher consistency among methods was observed on single-cell resolution datasets. We also saw little or no correlation among all methods on some datasets, highlighting algorithmic challenges due to the heterogeneity and complexity of real datasets. We further looked into the detailed trends of ranking consistency on top significant ctSVGs (from top 1 until top 200) with the CAT metric (Fig. 2D). Notably, all methods appeared to have a high concordance for the most significant ctSVGs but often not so for lower-ranked genes, which may reflect prominent spatial pattern variations on relatively few genes in these datasets (Fig. 2D, Figs S6–S10).

### Predictive performance of cell-type-specific spatially variable gene detection methods

**Figure 3:**
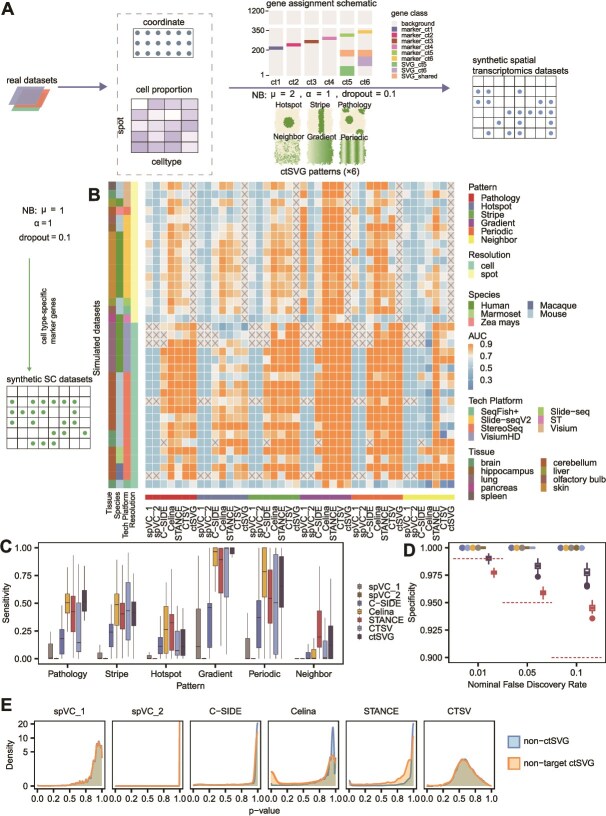
Benchmarking on simulated data. (A) Simulation strategy. Spatial coordinates and co-localization cell proportions for all spots were derived exactly from real datasets. Simulated gene expression counts (number of genes = 1200) were generated using a negative binomial distribution (mean = 2, dispersion = 1) with a 10% dropout rate. We retained the six major cell types (ct1 to ct6). Each cell type was assigned 25 marker genes. Only the two most abundant cell types (ct5 and ct6) were assigned ctSVGs, 75 unique in each and 50 shared by both, according to the six specified spatial variation patterns. The corresponding single-cell datasets were generated with a negative binomial distribution (mean = 1, dispersion = 1) and a 10% dropout rate. (B) AUC values of each method across the 37 simulated datasets with a 10% dropout rate, grouped by six spatial variation patterns. (C) Boxplot of sensitivity grouped by six spatial patterns. (D) Boxplot of specificity at different significance thresholds (.01,.05, and.1). (E) Density plot of gene *P*-value in one of the less abundant cell types (ct4) for 200 ctSVGs (ct5- or ct6-specific) versus 1000 non-ctSVG genes. Notice that for ct4, none of these genes are ctSVGs. This *P*-value is from all spot level datasets with a 10% dropout rate.

Due to the lack of gold-standard datasets with background truth, we simulated spatial transcriptomics data with known ctSVGs using location information and cell-type proportions from 37 real datasets, covering six distinct spatial gene expression patterns and including data at both spot-level and single-cell resolutions (Fig. 3A, Materials and Methods). In each synthetic dataset, six major cell types (ct1 to ct6) were simulated, with ctSVGs assigned to the two most abundant cell types (ct5 and ct6) but not assigned to the other, less abundant cell types (ct1 to ct4). In addition, cell-type marker genes were assigned to each cell type, respectively (Fig. 3A). In addition, reference single-cell datasets were also simulated.

For spot-resolution data, Celina and STANCE generally outperformed other methods in terms of overall AUC. In the Gradient pattern, spVC_1 and CTSV also performed well (Fig. 3B). Regarding single-cell resolution data, Celina, CTSV, ctSVG, and STANCE generally showed good performance across spatial patterns. In addition, Celina appeared to be best on the Hotspot pattern, whereas STANCE showed a clear advantage on the Neighbor pattern. The trend was largely reproducible upon different dropout rates (Figs S11 and S12). Nonetheless, in certain datasets such as ST_PDAC, most methods tended to be poorer in performance, suggesting that spatial distributions of cell-type proportions could have a potential impact on ctSVG detection.

AUC summarizes overall performance, but finer details are revealed by sensitivity and specificity. At a significance level of 0.05, Celina achieved the highest sensitivity on Stripe and Periodic patterns, whereas STANCE performed best on Hotspot and Neighbor patterns (Fig. 3C). ctSVG also performed well, especially on Pathology and Gradient patterns. CTSV turned out to be less stable, especially on the Periodic pattern. In contrast, spVC_1 and spVC_2 both showed a low sensitivity on nearly all spatial patterns, with spVC_1 slightly better. Subsequently, we assessed the false positive controllability by randomly permuting 100 times the spot coordinates of a real dataset (SeqFish+_cortex), thereby generating 100 permutated datasets with spatial structure completely eliminated, and no ctSVGs should be detected. Although all methods performed well, STANCE had a slightly lower specificity than other methods (Fig. 3D).

A major challenge in ctSVG detection is to deconvolute the confounding effects of spatial variations from different cell types. Therefore, we further investigated whether colocalization of cell types affects ctSVG detection on the target cell type, leading to potential false discoveries. We looked into *P*-values of 200 nontarget ctSVGs from more abundant cell types (ct5/6, gene identifiers 1–200 in Fig. 3A) in the less abundant target cell type (ct4), in comparison with 1000 non-ctSVGs in any cell type (gene identifiers 201–1200 in Fig. 3A). Notice that none of these two sets of genes are ctSVGs in the target cell type (ct4), and their *P*-values should ideally both distribute away from the significant end, i.e. near zero on the *P*-value axis (Fig. 3E). As expected, all methods produced distributions away from the zero end for non-ctSVGs. For nontarget ctSVGs, only Celina exhibited a small peak at $P = 0$, indicating the presence of false-positive predictions influenced by other cell types. These excessive false predictions remained upon increasing target cell proportions (Fig. S13) or replacing estimated cell-type proportions with true proportions (Fig. S14), suggesting their origination from the Celina model.

In addition, we also assessed the potential influences of upstream cell-type deconvolution on algorithmic performance. Using true cell-type proportions as a reference, we tested whether RCTD deconvolution estimates would change the relative performance of algorithms (Fig. S15). Overall, the deconvolution step had a minimal impact on the relative ranking of the methods. In most cases, the rankings remained unchanged or shifted by at most one or two positions. Overall, Celina again achieved the best AUC while STANCE also performed well, especially on the Neighbor pattern.

### Rotation invariance of cell-type-specific spatially variable gene detection methods

**Figure 4:**
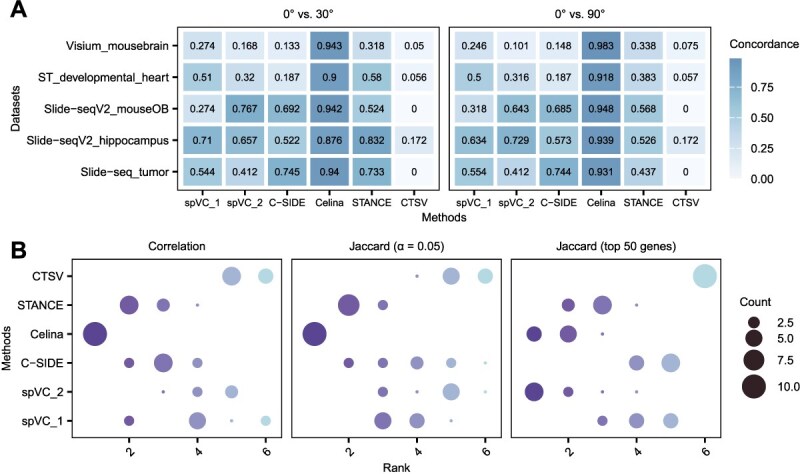
Evaluation of rotation robustness. (A) Heatmap showing the mean CAT scores (top 30 genes) upon two rotations (30 $^{\circ }$ and 90 $^{\circ }$) across the five spot level datasets. (B) The times count of each method being ranked by one of three robustness metrics, namely, the Spearman’s correlation (on *P*-value ranks) and the Jaccard index (either on genes defined by a significance level of 0.05 or top 50 ranked by *P*-value). For instance, with Spearman’s correlation metric, Celina was ranked as the top 1 for all 10 times.

Naturally, ctSVG detection should satisfy the property of rotation invariance, since rotation of the slice would not make any difference in the spatially varying expression of a gene. However, not all ctSVG algorithms have inherently satisfied this requirement, and the results might be sensitive to slice orientation [36, 60]. To evaluate this, we rotated five spot level real datasets to two different degrees (30$^{\circ }$ and 90$^{\circ }$), and then compared them with the original orientation on ctSVG identification (Fig. 4). Celina showed the highest rotation robustness, with an average CAT (top 30) above 0.87 (Fig. 4A). It also achieved the best performance in terms of Spearman’s correlation of *P*-value rankings and the Jaccard index at a significance level of 0.05 (Fig. 4B). STANCE performed well on some datasets but poorly on others, indicating strong dataset dependency (Figs S16–S20). CTSV consistently had the poorest rotation invariance, which is likely attributable to its additive and independent approximation of spatial effects along the two coordinate axes in each dimension. Strictly speaking, no methods perfectly achieved rotational invariance on spot or single-cell resolutions (Figs S21–S26). We noticed that STANCE claimed to be rotation-invariant [36, 60], which was not fully supported in our simulation results. To understand how this happened, we looked into the STANCE source code and found that this issue arose from its scaling strategy. Theoretical analysis in the STANCE paper ignores the scaling step, which in practice is sensitive to rotation that changes the range of coordinates and consequently the scaling factor (Fig. S27). This inconsistency thus alters the inter-spot Euclidean distances, thereby affecting the Gaussian kernel matrix and inferences.

### Scalability of cell-type-specific spatially variable gene detection methods

**Figure 5:**
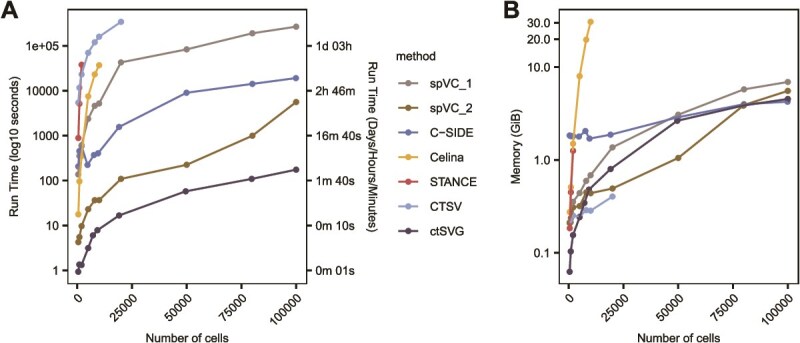
Evaluation of scalability. (A) Runtime statistics across datasets with varying numbers of cells/spots (fixed at 100 genes). (B) Peak memory usage statistics for datasets with varying numbers of cells/spots (fixed at 100 genes).

Constant technological evolution has been producing larger spatial transcriptomics datasets with increasing resolution. To confront this challenge, algorithmic scalability becomes highly crucial. We thus evaluated whether the ctSVG methods can deliver analysis results within reasonable time and memory limits. We allocated one CPU core to each method and set a maximum runtime of one week; results exceeding this time limit were excluded from the plots. In terms of efficiency, ctSVG had the best performance with the shortest CPU runtime, followed by spVC_2 (Fig. 5A). As the dataset sizes increased to 100 000 cells, ctSVG, spVC_2, and C-SIDE were still capable of maintaining stable runtime under 6 h, whereas CTSV, STANCE, and Celina experienced super-exponential increases in time, potentially limiting their practical use in high-resolution transcriptomic analysis. Moreover, Celina also showed a strong consumption of computational memory, exceeding 30 GB for a dataset of 100 genes and 10 000 cells (Fig. 5B). The other methods consumed <10 GB of memory even for datasets with up to 100 000 cells. In addition, STANCE and Celina frequently triggered errors for larger spatial data when the number of spots/cells exceeded 8000 and 20 000, respectively. Hence, future efforts are still highly warranted to develop more efficient computation frameworks in order to fully keep pace with the continuously increasing size and resolution of spatial transcriptomic data.

### A comparative case study on a lung cancer Visium HD data

**Figure 6:**
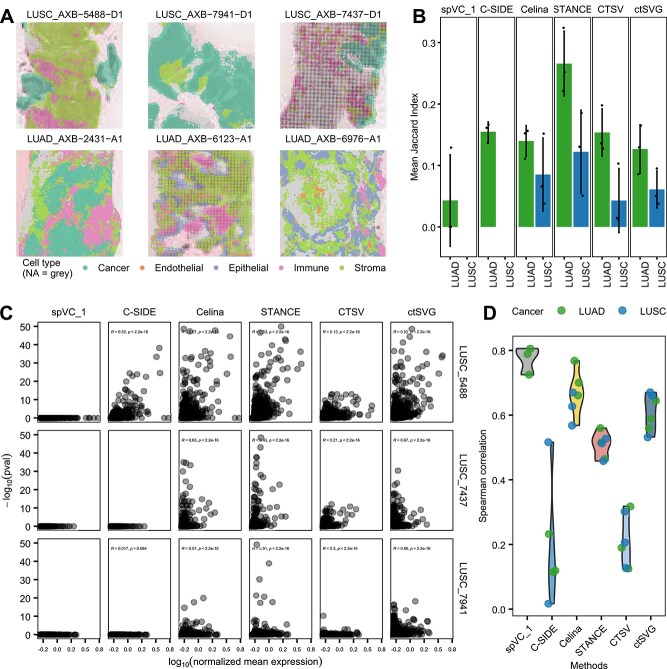
Evaluation on the pretreated lung cancer Visium HD data. (A) Six dissected tissue sections from LUSC and LUAD patients, overlaying with the corresponding cell-type distributions. Shown are representative portions. Gray: spots with unknown cell-type composition. See Table S1 for detailed patient information. (B) Jaccard similarity indices of ctSVGs obtained by the same method across the three patients within the same lung cancer subtype. (C) Scatter plots with Spearman’s correlation coefficients between *P*-values and expression levels in the three LUSC sections, grouped by each method. (D) Violin plot summarizing the Spearman’s correlation coefficients between *P*-values and expression levels across the six sections, grouped by each method.

To assess the capacity of different methods to detect conserved versus patient-specific heterogeneous genes, we performed Visium HD spatial transcriptomics on biopsies from six pretreatment lung cancer patients (Materials and Methods), including three lung adenocarcinoma (LUAD) and three lung squamous cell carcinoma (LUSC) (Fig. 6A, Figs S28 and S29, Table S1). Here, we focused on the ctSVGs specific to cancer-associated fibroblasts (CAFs). STANCE identified the largest proportions of conserved ctSVGs in both LUAD and LUSC, which were mainly enriched in pathways associated with invasive tumor microenvironment states (Fig. 6B, Figs S30 and S31). In addition, there were also functional differences of conserved significant ctSVGs by different methods (Fig. S31). For instance, Celina and STANCE distinctively revealed deregulation of cell and cell–substrate adhesion pathways in conserved LUAD ctSVGs, whereas ctSVG and CTSV uniquely identified mucosal immune response. Interestingly, we also noticed a greater heterogeneity of CAF ctSVGs in LUSC than in LUAD, which could be potentially related to a stronger extracellular matrix (ECM) remodeling in LUSC [61].

We further examined the relationship between ctSVG significance and gene expression levels. spVC_1, Celina, ctSVG, and STANCE showed a stronger correlation than all the other methods (Fig. 6C and D, Fig. S32). Although potential algorithmic or data sparsity factors might increase the detection rate of highly expressed genes, we observed that the candidate genes exhibited significant enrichment in pathways associated with cancer progression and fibroblast activity, encompassing critical processes such as immune modulation, ECM remodeling, and tumor-associated invasion and metastasis (Fig. S33). Nonetheless, future evaluation would be helpful to further dissect the relative contributions of biological and technical factors.

### A comparative case study on a melanoma Slide-seqV2 dataset

**Figure 7:**
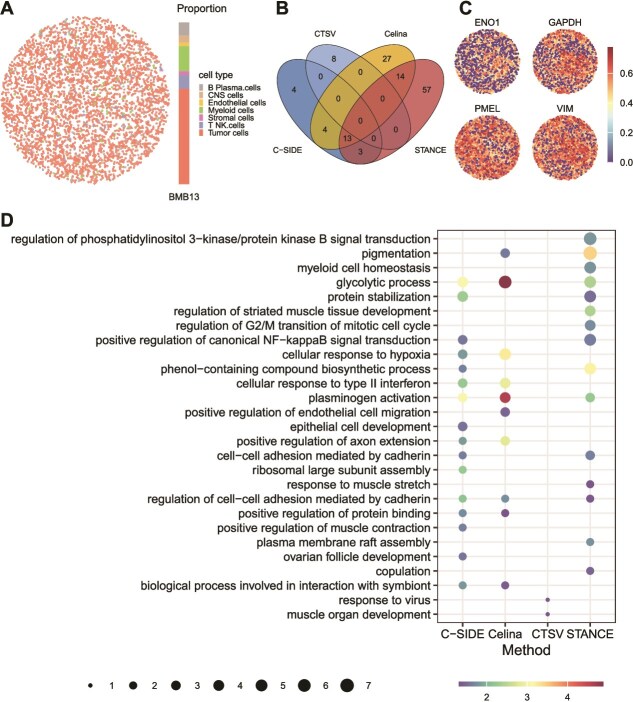
Evaluation on an untreated melanoma slide-seqv2 dataset [62]. (A) Cell type distribution on the MBM13 slice. Left: spatial distribution of cell types. Right: proportion of each cell type. (B) Overlap of ctSVGs identified as significant by the four methods applicable to this dataset. (C) Spatial expression patterns for the four ctSVGs commonly detected as in the top 7 by STANCE, C-SIDE, and Celina. (D) GO biological process (BP) pathways enriched in the significant ctSVGs by each method. GO BPs enriched in at least one method were shown. Dot color: pathway significance; dot size: number of enriched genes.

We also performed a comparative study by analyzing a public melanoma dataset on the Slide-seqV2 platform with accompanying single-nucleus RNA sequencing (snRNA-seq) data [62]. RCTD deconvolution revealed that tumor cells were the most abundant cell type for ctSVG analysis (Fig. 7A). Consistent with previous results, Celina and STANCE identified more ctSVGs, while C-SIDE and CTSV detected fewer, whereas spVC_2 and spVC_1 detected none. Celina, C-SIDE, and STANCE shared thirteen overlapping genes, with five overlapping genes among their top ten results. CTSV detected eight significant genes, all of which were unique. The top four most highly ranked overlapping genes are shown in Fig. 7C. Similar patterns were observed in the two glycolysis genes (ENO1 and GAPDH) and the epithelial-to-mesenchymal transition (EMT) marker (VIM), consistent with the known metabolic reprogramming of EMT in cancer cells [63]. Interestingly, these genes, along with the melanocyte-specific gene (PMEL), exhibited a spatially dichotomous pattern of expression, suggesting an remarkable local heterogeneity in melanoma tumor cells. Although some methods identified several consensus and biologically interpretable ctSVGs, we still observed notable differences in inferred spatially variable pathways based on ctSVGs from each method (Fig. 7D). For instance, glycolysis ranked variable across methods, ranging from top 1 to $\sim$17th (Table S3). Moreover, the distinct pathways enriched by different methods also highlighted different biological interpretations. For instance, C-SIDE specifically identified EMT-related pathways such as epithelial cell development, Celina specifically identified hypoxia-related vascular functions such as endothelial cell migration, whereas CTSV identified no common pathways with other methods. These differences underscore that method-specific modeling and ctSVG identification could lead to distinct biological interpretations.

### Suggestions for choosing cell-type-specific spatially variable gene methods

The results of this study suggest practical considerations on methodological choice when performing ctSVG analysis. For datasets with >5000 spatial spots or under hardware constraints (CPU or memory), spVC_1/2, ctSVG, or C-SIDE are recommended for their higher computation efficiency. For single-cell resolution data, ctSVG is recommended for good performance and high efficiency, whereas spVC is not recommended as it could exhibit reduced efficacy due to potential identifiability constraints, as noted in the original study [34]. When sensitivity is a top requirement, STANCE, Celina, and ctSVG are good choices. When tighter control of false positives is warranted, C-SIDE, spVC_1, ctSVG, and CTSV could be considered as reasonable choices.

## Discussion

To our knowledge, this work represents the first comprehensive evaluation of ctSVG detection algorithms. We systematically examine the computation behavior of these methods under a range of biologically and technically motivated scenarios, including real datasets of different platforms and resolutions, real-data-grounded large-scale simulations, rotation-invariance analyses, different types of false-positive controls, and comparative illustration of biological interpretability across methods. Together, these efforts offer a more complete picture of ctSVG algorithmic performance. To improve transparency, we have summarized the source datasets used for each figure in Table S2, and deposited all the source codes on GitHub.

**Figure 8:**
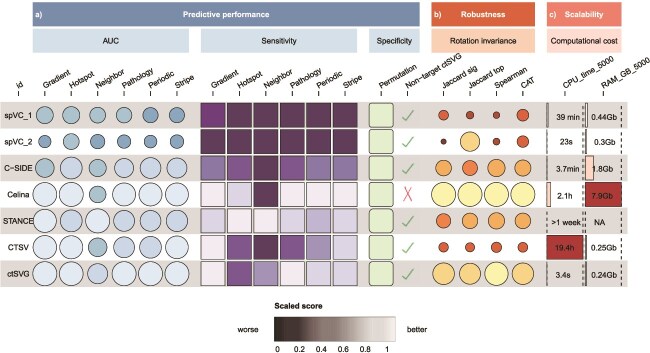
Summary of benchmarking performance in terms of predictive performance, robustness, and scalability. For predictive performance and robustness, lighter colors and larger areas indicate better performance. For scalability, computational costs were assessed on datasets with 5000 cells, where darker colors and longer bars represent higher resource consumption. Jaccard sig/top: Jaccard similarity of significant/top genes. NA: not available.

ctSVG detection approaches integrate cell-type proportions and spatial expression information, offering strong potential for in-depth molecular characterization of tissue heterogeneity and plasticity. In this study, based on 46 real datasets, 666 simulated datasets on designed conditions, 10 rotation datasets, and 100 permutation datasets, we provided an in-time comprehensive evaluation of current state-of-the-art methods in terms of consistency, predictive performance, robustness, scalability, and biological interpretability (Fig. 8). This should assist users in the adoption of methods and highlight directions for future algorithmic development.

We found that the methods based on the spline basis function tended to have a lower detection power compared with the methods based on the kernel. Although increasing the number of basis functions could improve spatial granularity by allowing finer local variation, their sensitivity seems to be lower than the kernel-based methods, possibly due to the limited structural flexibility of predefined basis functions when compared with kernel methods [64, 65]. Nonetheless, the methods based on spline functions showed clear advantages in computational efficiency. These findings underscore that the selection of model architecture has a significant impact on algorithm performance, suggesting that future methodological efforts should aim to balance the characteristics of different model architectures.

In addition to the overall choice of model architectures, we showed that methodological details also strongly influenced their applicability. The first unexpected finding was that STANCE, which claims to be rotation-invariant, remained affected by rotation in our benchmark. This discrepancy arose because the original authors considered the rotational invariance of the Gaussian kernel but overlooked the effect of scaling on Euclidean distances. Since scaling of spatial coordinates is a common preprocessing step in spatial transcriptomics analysis [35, 36, 66, 67], future methodological development should explicitly account for such scaling effects to minimize their impact on model performance. Our second noteworthy observation was that Celina exhibited more false positives as influenced by colocalization of nontarget cell types. This may be because Celina models spatial correlation solely through random effects assigned to the target cell type. When other cell types display spatial structures that are not sufficiently explained by fixed effects or random effects, the residual spatial signals may be incorrectly attributed to the target cell type. The third phenomenon we observed is the relatively insufficient statistical power of spVC_2 (two-step), which might originate from its two-step testing. Its latter step depends on the filtered genes by the first step, thereby assuming only SVGs can be ctSVGs and leading to the false removal of a subset of true ctSVGs. The fourth observation is that methods such as CTSV, STANCE, and Celina are restricted by their computation resource requirements for large or high-resolution datasets. Overall, more flexible model assumptions and highly efficient computation frameworks will be needed in future methodological development.

Apart from the deficiencies of model assumptions, uneven cell-type composition introduces additional challenges in spatial transcriptomics analysis. Dominant cell types benefit from greater transcript abundance, making it easier to identify ctSVGs, whereas the sparse spatial distribution of rare cell types limits the ability to capture their expression patterns comprehensively. Addressing this issue requires not only methodological advances to reduce bias and improve sensitivity but also technological improvements in spatial transcriptomics platforms. Enhancing transcript capture efficiency and dropout will be critical to accurately characterizing rare cell populations. Thus, future developments in spatial transcriptomics should aim for robust and accurate detection of gene expression at true single-cell resolution with reduced dropout rates [50, 68, 69].

Our study also has some limitations. There is still a lack of widely accepted gold-standard real datasets with reliable ground truth. While the simulated data have such information, they may not fully embrace an equivalent level of complexity, such as diverse cell-type compositions, spatial distributions, and transcript capture efficiencies.

Key Points

- We present the first comprehensive benchmark of six cutting-edge cell-type-specific spatially variable gene (ctSVG) detection methods.
- We generate a new high-resolution Visium HD dataset of six lung cancer patients for benchmarking.
- STANCE and Celina excel in predictive performance, whereas C-SIDE, spVC, ctSVG, and CTSV have better control of false positives.
- Rotation invariance, false positive control, and computational scalability remain key issues for future algorithmic design.
- Algorithm choice substantially affects downstream biological interpretations.

## Supplementary Material

bbag190_Supplemental_Files

## Data Availability

The newly sequenced lung cancer dataset has been uploaded to the OMIX database of the National Genomics Data Center (https://ngdc.cncb.ac.cn) under the accession number OMIX012132 (project PRJCA047286). This will be released after the publication of this paper. All benchmarking codes are available on a GitHub repository at https://github.com/FangZY-Lab/ctSVGbench.
