# Supplementary material for: Benchmarking cell-type-specific spatially variable gene detection methods
Source: Brief Bioinform. 2026 Apr 27;27(2):bbag190. doi: 10.1093/bib/bbag190 (PMC13112436; doi:10.1093/bib/bbag190)
Supplement: bbag190_Supplemental_Files [file bbag190_supplemental_files.zip › SI_bbag190.pdf]

Supplementary file for “Benchmarking cell-type-specific spatially variable genes detection methods”

Hui Yao[1†], Shuai Mu[2†], Fei He[3], Zhaoyuan Fang[1,4*]

[1]Department of Colorectal Surgery and Oncology of the Second Affiliated Hospital, and Centre of Biomedical Systems and Informatics of Zhejiang University-University of Edinburgh Institute (ZJU-UoE Institute), Zhejiang University School of Medicine, Zhejiang University, 866 Yuhangtang Road, Xihu District, Hangzhou, 310058, Zhejiang, China.
[2]Senior Department of Oncology, The First Medical Center, Chinese People’s Liberation Army (PLA) General Hospital, 28 Fuxing Road, Haidian District, Beijing, 100039, China.
[3]Shanghai Neo-Biotechnology Co., Ltd, 518 XinZhuan Road, Shanghai, 201612, Shanghai, China.
[4]Edinburgh Medical School, College of Medicine and Veterinary Medicine, The University of Edinburgh, Edinburgh, United Kingdom.

*Corresponding author(s). E-mail(s): fangzhaoyuan@sibs.ac.cn; zhaoyuanfang@intl.zju.edu.cn;
Contributing authors: hui1.23@intl.zju.edu.cn; mushuai_yige@126.com; hefeistat@qq.com;
†These authors contributed equally to this work.

A1 Supplementary methods

A1.1 Collection of public datasets

The 40 publicly available spatial transcriptomics datasets differed greatly in size, with some including tens of thousands of spatial spots that could challenge the computational capacity of certain analysis methods. For datasets with multiple tissue sections, only one representative section was selected (see Table S2 for details). To further

ensure fair and efficient benchmarking, large datasets were cropped and subsetted to fewer than 3,000 spots. The subsetting process was performed by centering on the median spatial coordinates and iteratively adjusting the selection window size to include a region symmetrically around the center whose spot count closely matched the desired target, thereby retaining the representative central tissue structure while reducing data size for computational feasibility.

Slide-seqV2_melanoma [1] spatial data and paired scRNA-seq reference data are available from the Gene Expression Omnibus (GEO) under accession code GSE200218.

Slide-seqV2_hippocampus [2] spatial data are available from the Single Cell Portal under SCP948. The paired scRNA-seq data are available from GEO under GSE162631.

Slide-seqV2_mouseOB [3] spatial data are available from GEO under GSE169012. The paired scRNA-seq data are available from GEO under GSE108097.

Visium_skin [4] spatial data are available from GEO under GSE144239. The paired scRNA-seq data are available from GEO under GSE144236.

Visium_spleen [5, 6], spatial data are available from GEO under GSE200720. The paired scRNA-seq data are available from GEO under GSE109774.

Visium_bladder [7] spatial data are available from GEO under GSE171351. The paired scRNA-seq data are available from GEO under GSE169379.

Visium_liver [8, 9] spatial data are available fromGEO under GSE167096. The paired scRNA-seq data are available from ArrayExpress under accession code E-MTAB-7407.

Visium_intestine [10, 11] spatial data are available from 10x Genomics website. The paired scRNA-seq data are available from GEO under GSE132465.

Visium_tail [12] spatial data are available from GEO under GSE245313. The paired scRNA-seq data are available from GEO under GSE245312.

Visium_pancreas [13] spatial data are available from GEO under GSE244534. The paired scRNA-seq data are available from GEO under GSE243466.

MERFISH_hypothalamus [14] data are available from Dryad. The paired scRNA-seq are available from the GitHub repository.

seqFISH+_mouse_OB [15] data and paired scRNA-seq are available from the seqFISH+ GitHub repository.

ST_PDAC spatial [16] data and paired scRNA-seq data are available from GEO under GSE111672.

ST_developmental_heart [17] spatial data and paired scRNA-seq data are available from Mendeley Data

Visium_lymph_node [18] spatial data are available from 10x Genomics website.

Visium_mousebrain [19] spatial data are available from the 10x Genomics website. The paired scRNA-seq data are available from EBI under E-MTAB-11115.

Visium_melanoma [20] spatial data and paired scRNA-seq data are available from GEO under GSE159709.

Slide-seq_tumor [21] spatial data and paired scRNA-Seq data are available from the Single Cell Portal under SCP1663.

StereoSeq_mouseOB [22, 23] spatial data and paired scRNA-seq data are available from MOSTA.

StereoSeq_MDESTA [24] spatial data and the paired scRNA-seq data are available from MDESTA.

StereoSeq_CBMSTA_Marmoset [25] spatial data paired scRNA-seq data are available from CBMSTA.

StereoSeq_CBMSTA_Macaque [25] spatial data and paired scRNA-seq data are available from CBMSTA.

A1.2 Generation of synthetic datasets

We designed simulation experiments based on the spatial locations and cell type proportions at each location from real data. Expression counts for spatial (mean = 2, dispersion = 1) and single-cell (mean = 1, dispersion = 1) transcriptomics were both generated using a negative binomial distribution. To simulate the noisy events present in real sequencing data, we additionally introduced dropout events by randomly setting a portion of raw counts to zero at three levels of dropout rates (10%, 20%, and 30%).

Each simulated spatial transcriptomics dataset contains six cell types, named from cell type 1 (ct1) to 6 (ct6) in descending order of abundance. The total number of genes is 1,200, with 25 marker genes per cell type. The two most abundant cell types each contain 125 ctSVGs, 50 of which are shared. For the shared ctSVGs, each cell type has independent directions and magnitudes of fold changes to better reflect the biological complexity in real systems.

Six spatially varying patterns are designed for ctSVGs to represent those observed in research. The Hotspot pattern, with a central point randomly chosen, prescribes only the surrounding spots (cells) within a defined radius exhibiting expression changes in ctSVGs. The Stripe pattern defines fixed-width stripes along the x-axis, with ctSVG expression amplified within the stripes relative to the outside ones. In the Gradient pattern, ctSVG expression increased or decreased linearly along the x-axis, constituting continuous spatial gradients. The Neighbor pattern reflects microenvironmental influences from other cells, where ctSVG expression changes proportionally to the density of another cell type. In the Pathology pattern, multiple random centers define localized regions with altered expression in nearby spots (cells) as compared to more distant spots (cells), mimicking localized pathological features. Finally, the Periodic pattern simulates sinusoidal fluctuations along the x-axis, representing periodic or layered tissue arrangements. For all these patterns, expression at each spot was designated as the weighted combination of cell-type-specific mean expression by cell-type proportions, and the corresponding count was generated using a negative binomial distribution.

The patterns of spatial expression changes of ctSVGs can be either non-continuous (Pathology, Hotspot, and Stripe) or continuous (Gradient, Periodic, and Neighbor). To maintain relative uniformity of the total counts across spots (cells) without confounding the assigned expression changes, asymmetric numbers of up- (10) and down-regulated (190) genes were specified for non-continuous spatial patterns. For non-continuous ctSVGs, their fold changes are randomly chosen at discrete levels, with up-regulated genes randomly set to 5- or 10-fold, and down-regulated genes randomly

set to 0.25- or 0.5-fold. For continuous spatial patterns, fold changes vary continuously from 0 to 3 across spatial locations, and the direction of change differs among genes.

A1.3 Parameter settings for ctSVG methods

All analyses were performed in R (v4.4.3). The versions of the R packages used were: STANCE v0.3.0, CELINA v1.0, spVC v0.1.0, spacexr v2.2.1, ctSVG v1.0, and CTSV v1.8.0. We applied C-SIDE(implemented in the spacexr package) in its nonparametric mode with $cell\_type\_threshold = 0$ to include all cell types, ensuring consistency across methods. ctSVG supports both de novo cell type clustering and predefined cell types of single-cell spots. In this benchmarking, the latter approach was chosen for compatibility with other methods. All remaining configurations were left as the default. For CTSV, spVC, Celina, and STANCE, all settings were left as default.

A2 Supplementary tables

Supplementary Table S1: Patient and clinical information for the Visium HD lung cancer sections. Columns include lung cancer subtype, patient ID, gender, age, height, weight, smoking years, pathology, sequencing data ID, biopsy date, therapy date, and the pretreatment label.
Supplementary Table S2: Summary of the 46 real datasets used in the comparative analysis. Columns include the dataset name, number of spots (or cells), number of genes, technology platform, spatial resolution, species, tissue type, the evaluation section in which the dataset was used, paird project, and used sample.
Supplementary Table S3: Top-ranked Gene Ontology (GO) biological process enrichment results for ctSVGs identified by each method, with glycolysis-related pathways highlighted. The table includes the following columns: pathway ID, description, adjusted p-value, the method used, and rank.

A3 Supplementary figures

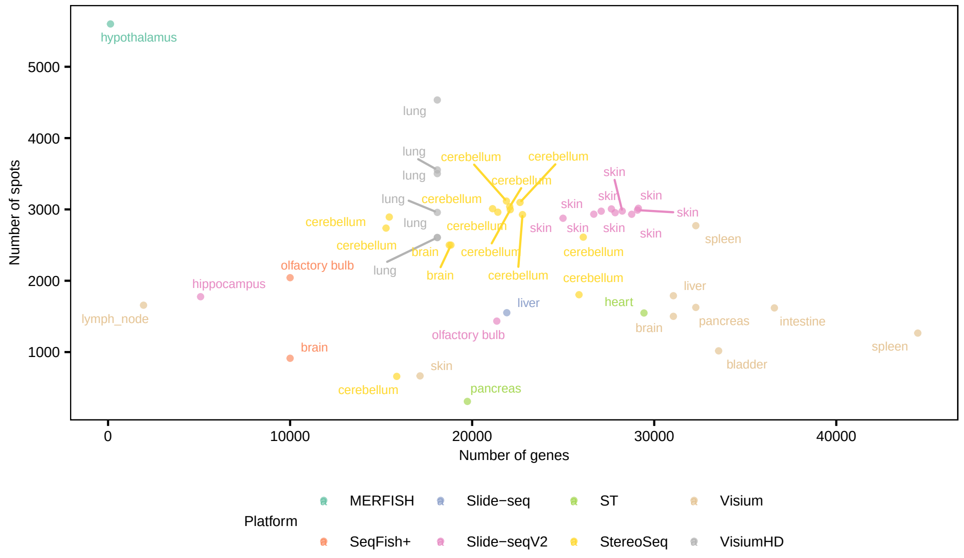

Fig. S1 Scatter plot showing the number of genes and spots(cells) in the 60 real datasets used, derived from 8 different platforms.

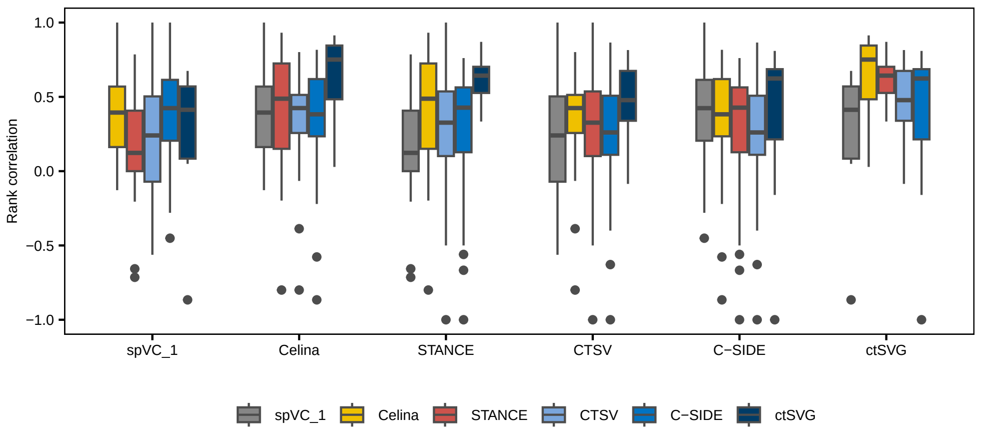

Fig. S2 Boxplot summarizing the pairwise rank correlations of adjusted p-values across all methods in both spot resolution and single-cell resolution datasets.

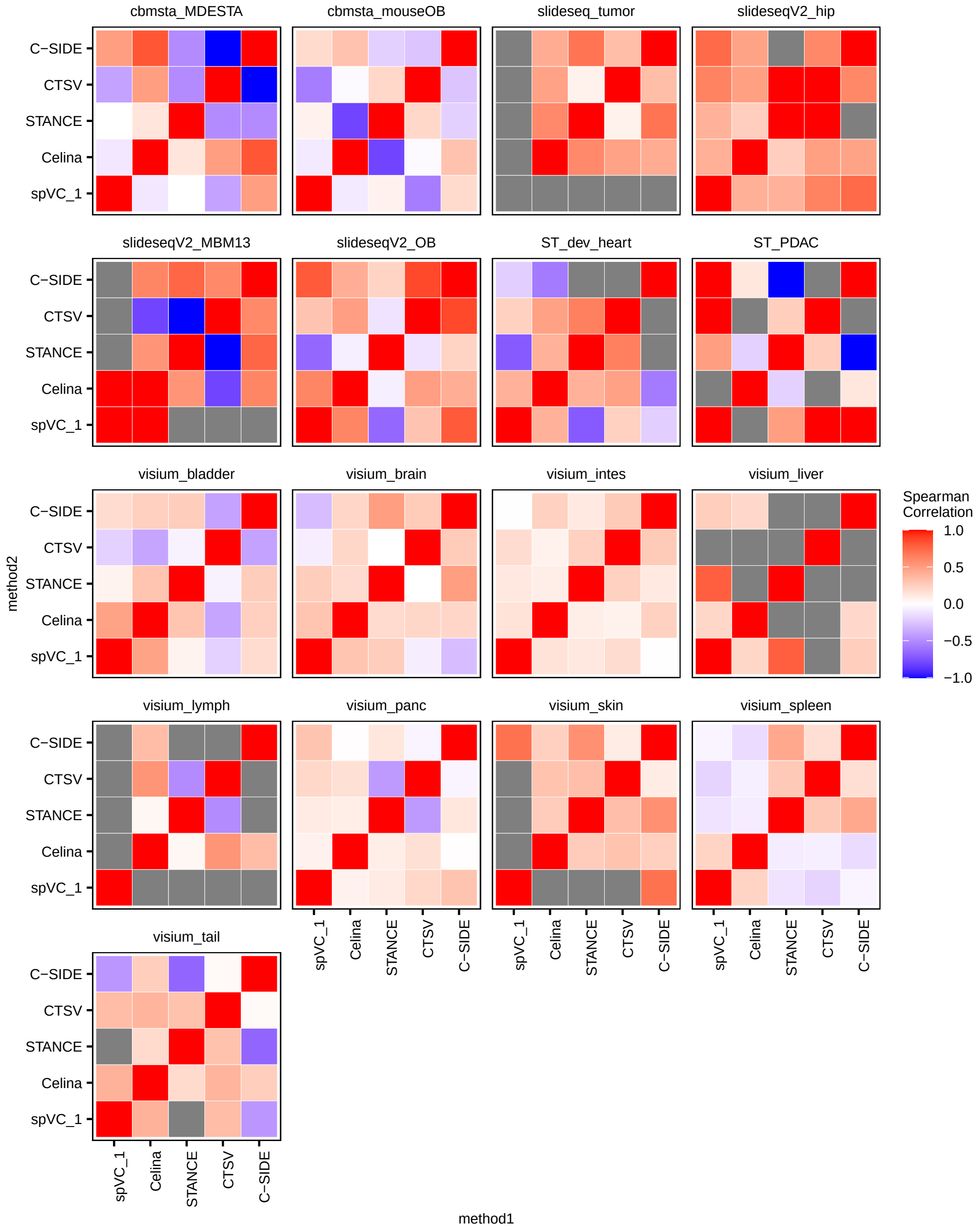

Fig. S3 Heatmap showing the pairwise rank correlations of adjusted p-values across methods within each spot level dataset.

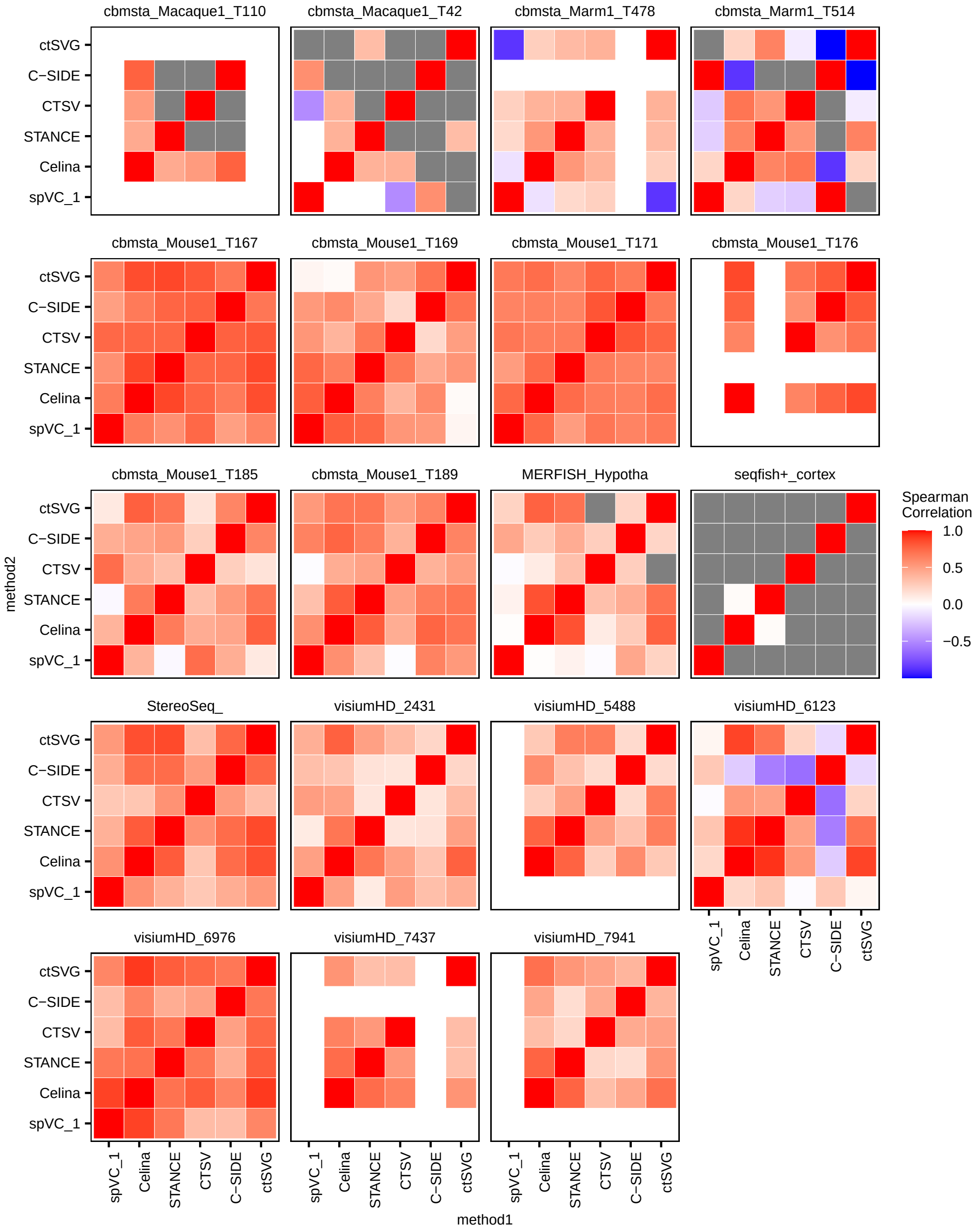

Fig. S4 Heatmap showing the pairwise rank correlations of adjusted p-values across methods within each cell level dataset.

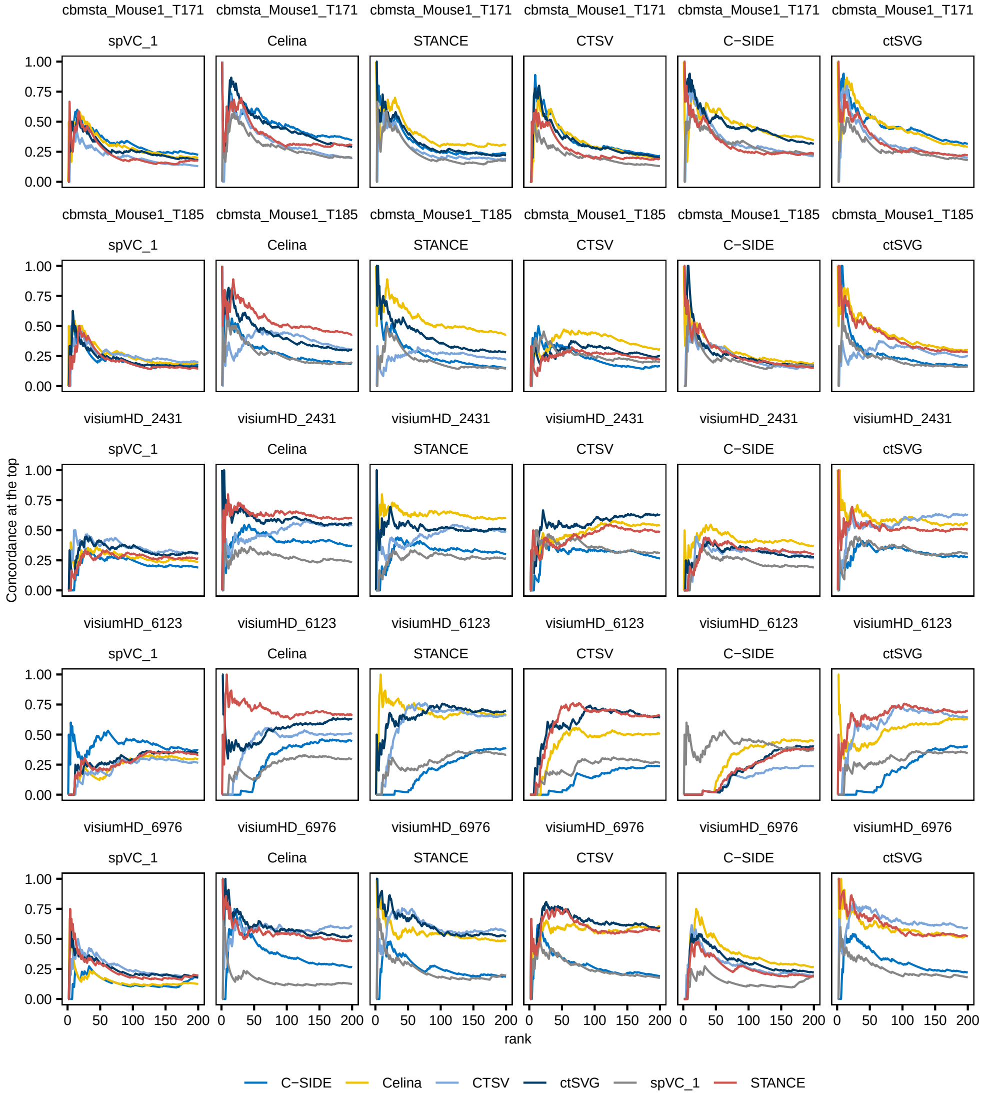

Fig. S5 Pairwise Concordance at the Top (CAT) (top 200 genes ranked by adjusted p-values) for the four datasets shown in the figure.

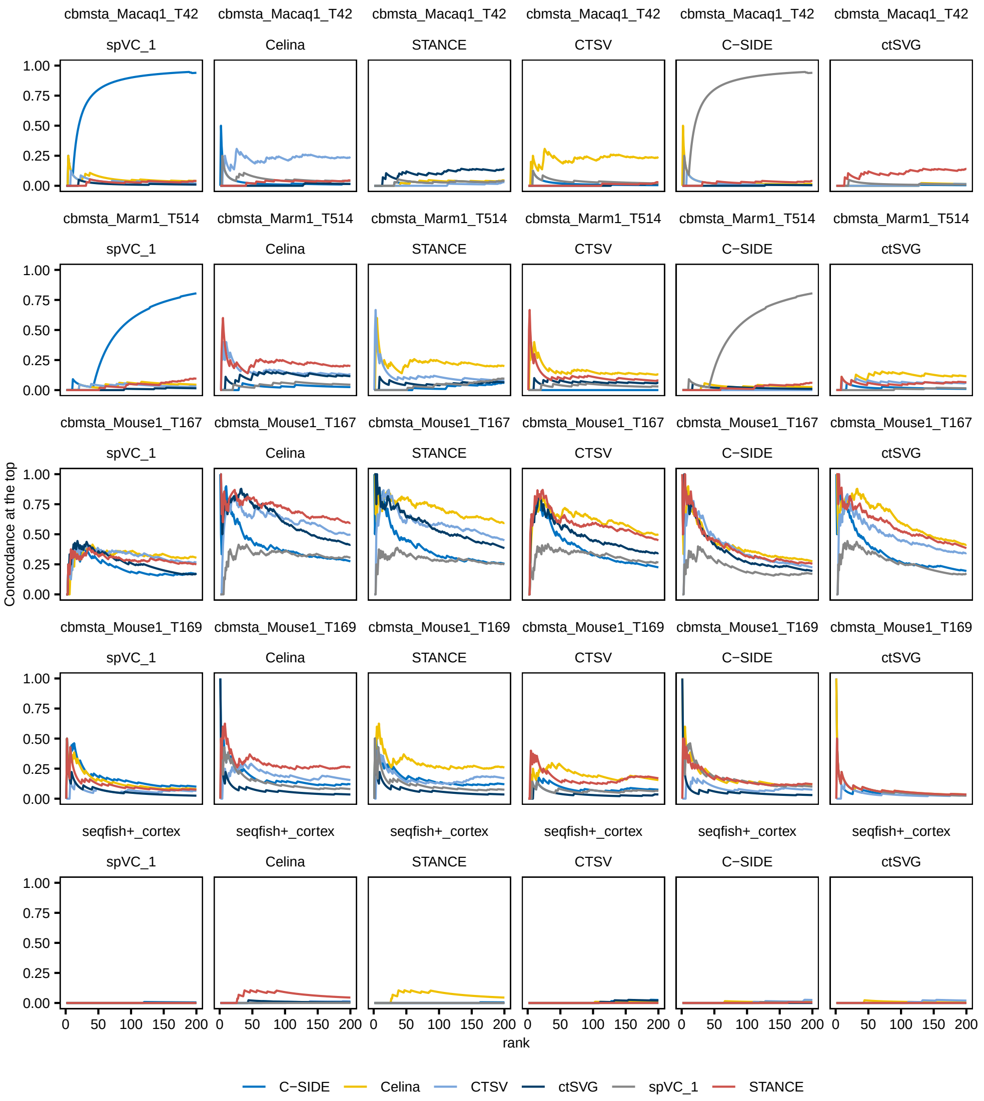

Fig. S6 Pairwise Concordance at the Top (CAT) (top 200 genes ranked by adjusted *p*-values) for the four datasets shown in the figure.

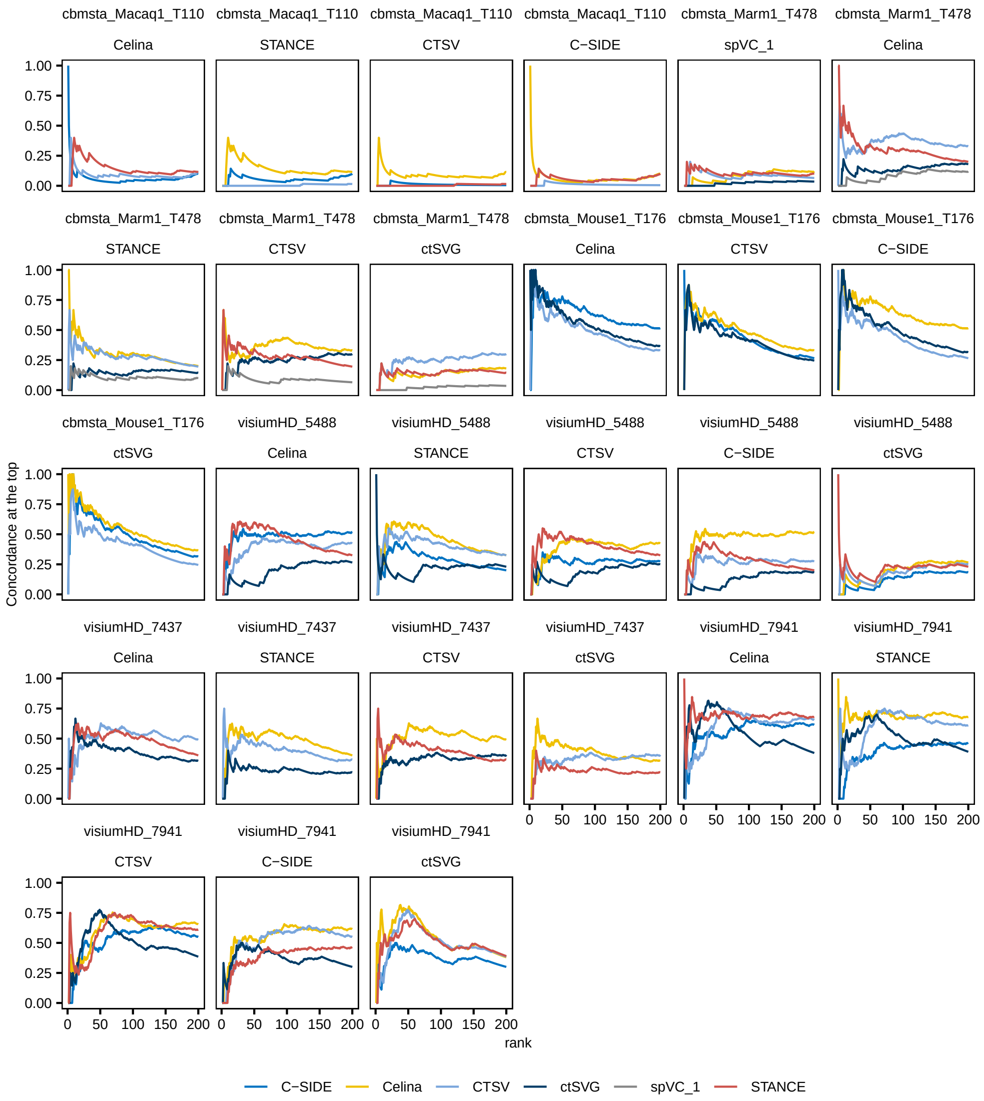

Fig. S7 Pairwise Concordance at the Top (CAT) (top 200 genes ranked by adjusted p-values) for the four datasets shown in the figure.

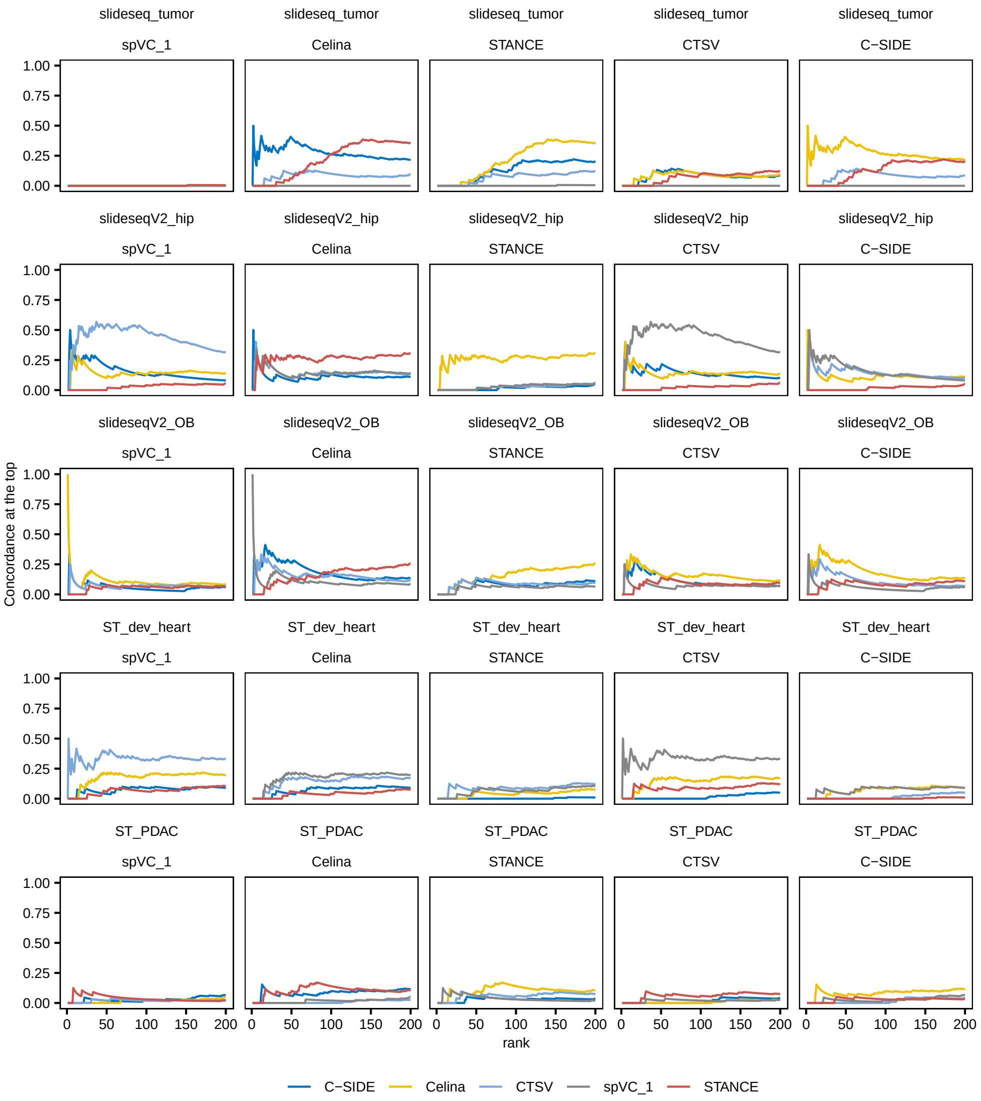

Fig. S8 Pairwise Concordance at the Top (CAT) (top 200 genes ranked by adjusted p-values) for the five datasets shown in the figure.

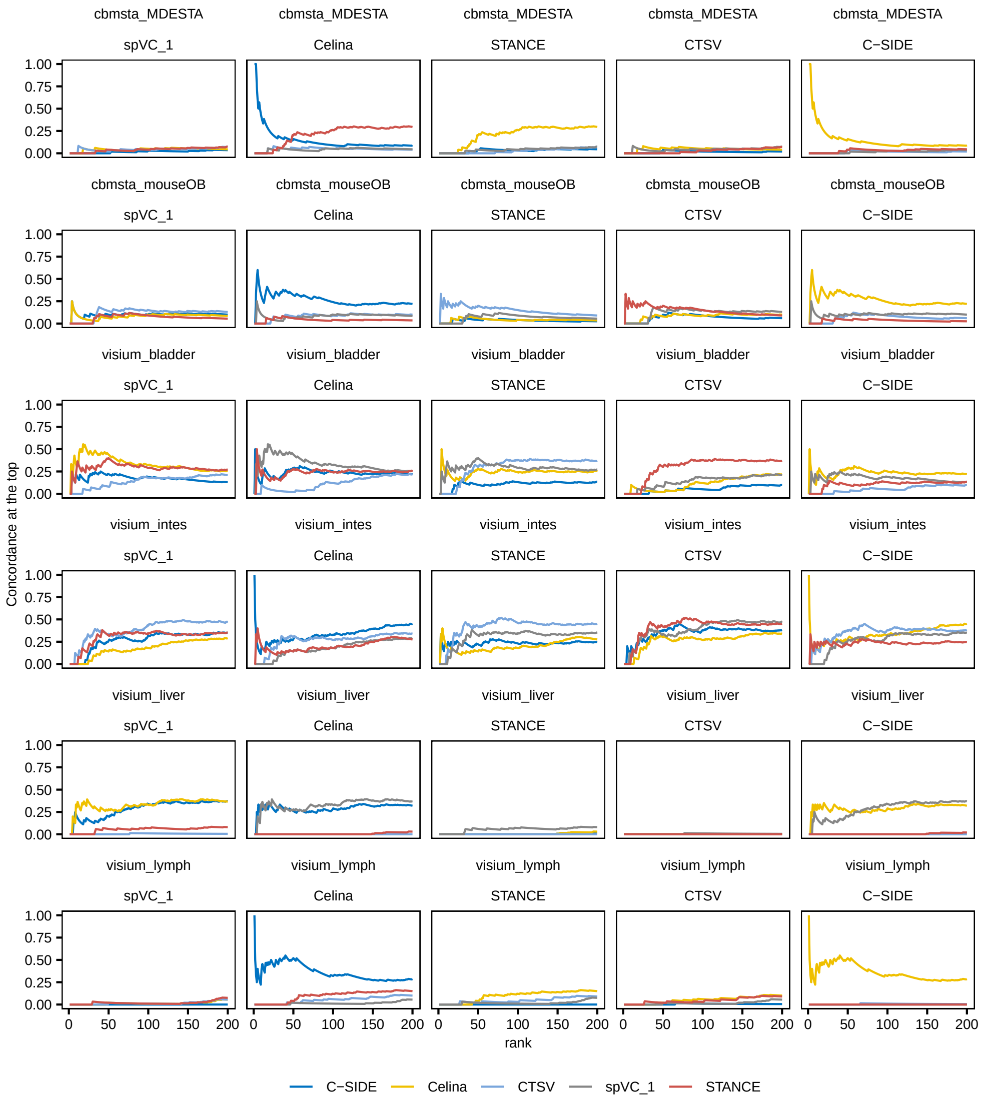

Fig. S9 Pairwise Concordance at the Top (CAT) (top 200 genes ranked by adjusted p-values) for the five datasets shown in the figure.

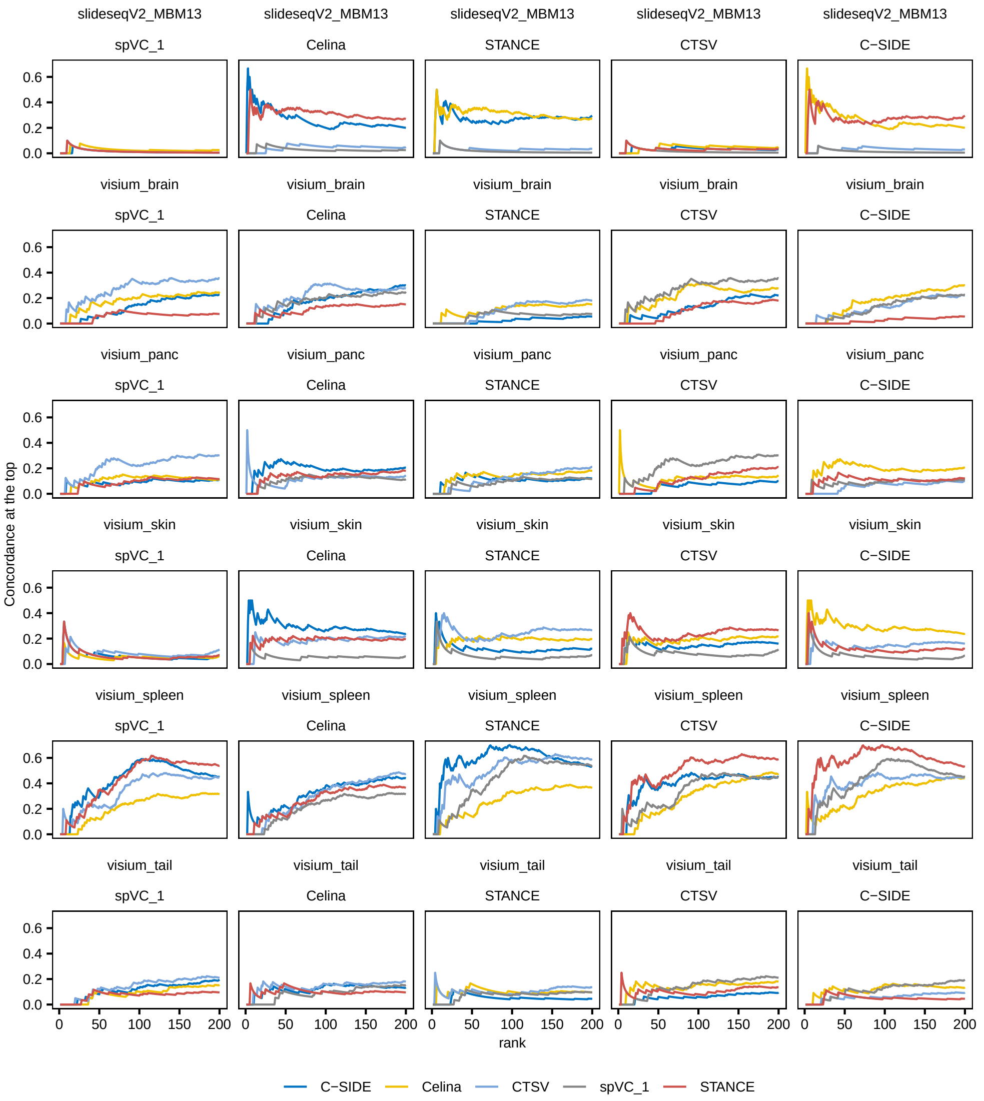

Fig. S10 Pairwise Concordance at the Top (CAT) (top 200 genes ranked by adjusted p-values) for the four datasets shown in the figure.

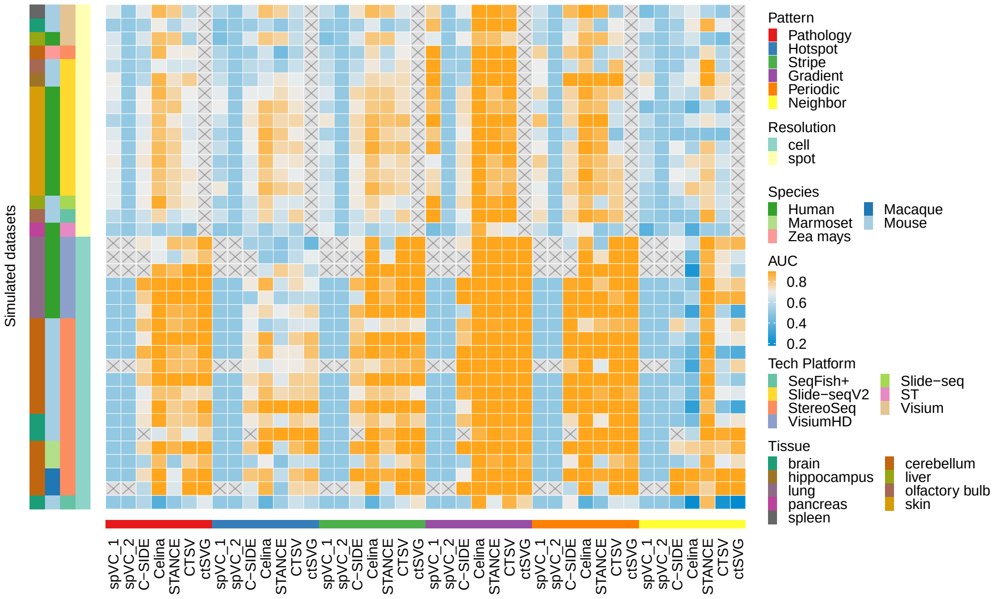

Fig. S11 AUC values of each method across the 37 simulated datasets with a 20% dropout rate, grouped by six spatial variation patterns.

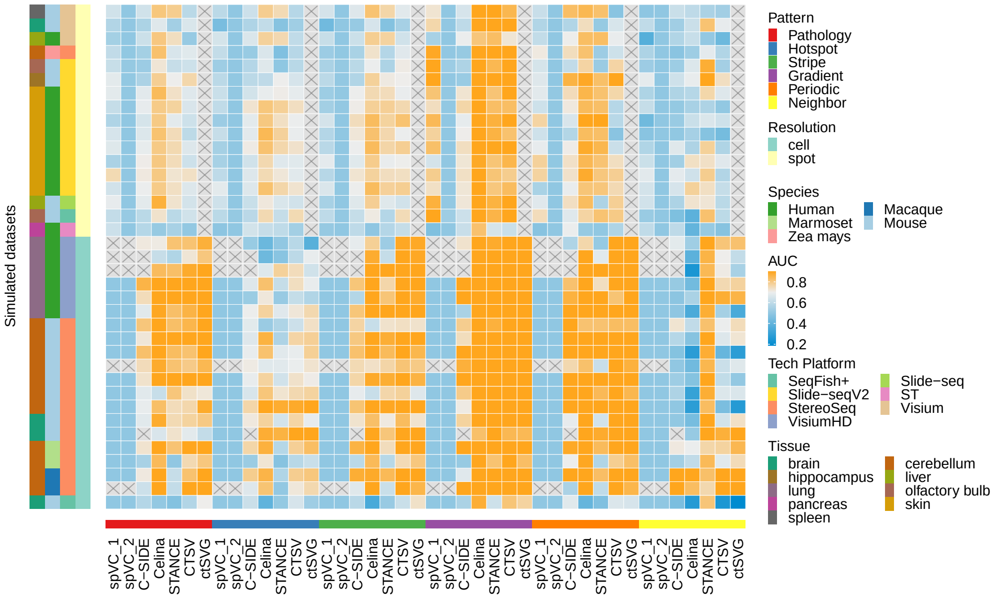

Fig. S12 AUC values of each method across the 37 simulated datasets with a 30% dropout rate, grouped by six spatial variation patterns.

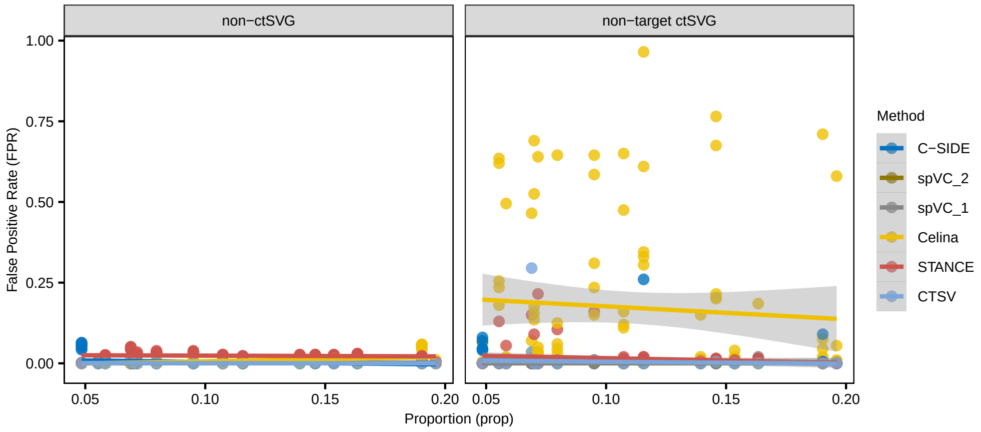

Fig. S13 Scatter plots show the proportion of cell type 4 across datasets and the corresponding false positive rates, with each color indicating a different method.

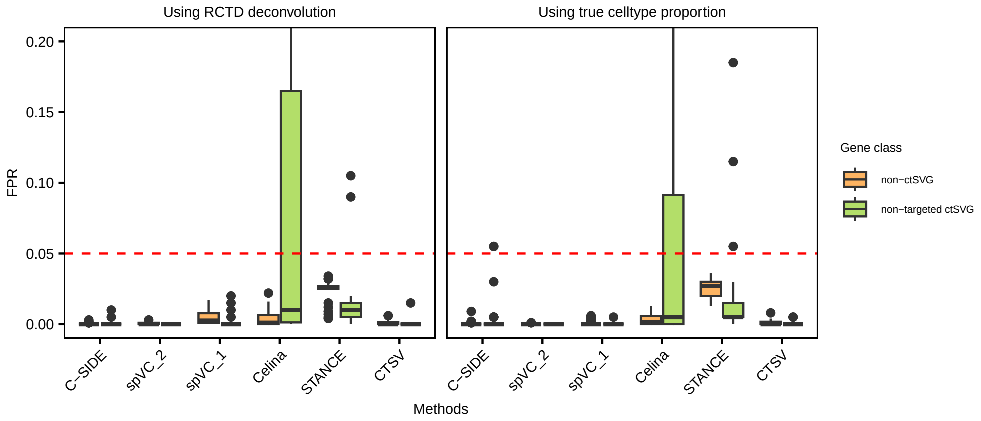

Fig. S14 Boxplots comparing the false positive rates of affected cell types in spot-level data using deconvolution versus directly inputting true cell-type proportions, with a red dashed line denoting a false positive rate of 0.05.

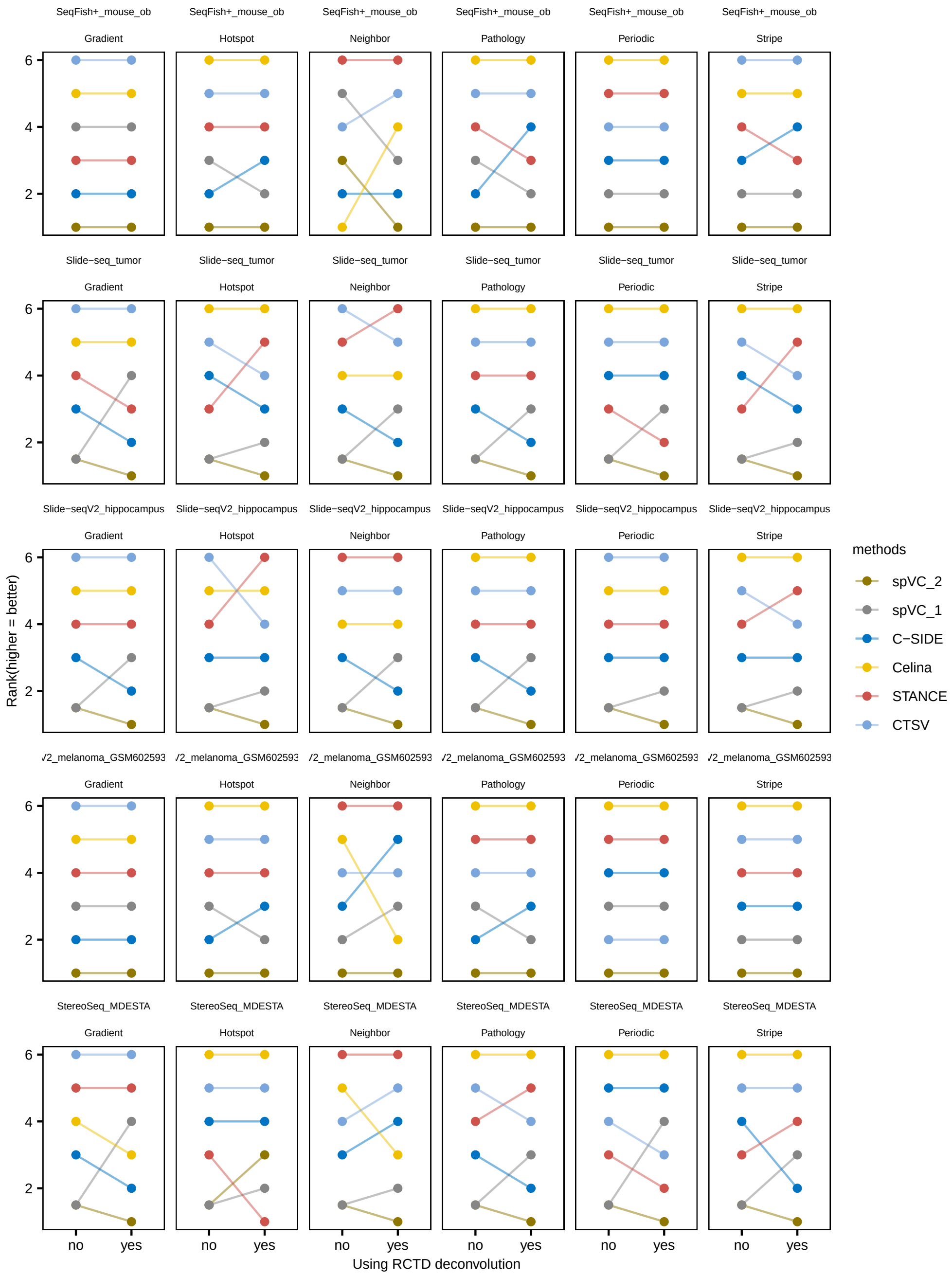

Fig. S15 AUC rankings for each method under different conditions. A higher AUC corresponds to a higher ranking, indicating better performance. Each color represents a method. The x-axis indicates whether a deconvolution method is applied, and the y-axis shows the ranking. Each row of panels corresponds to a dataset, and each column corresponds to a spatial pattern.

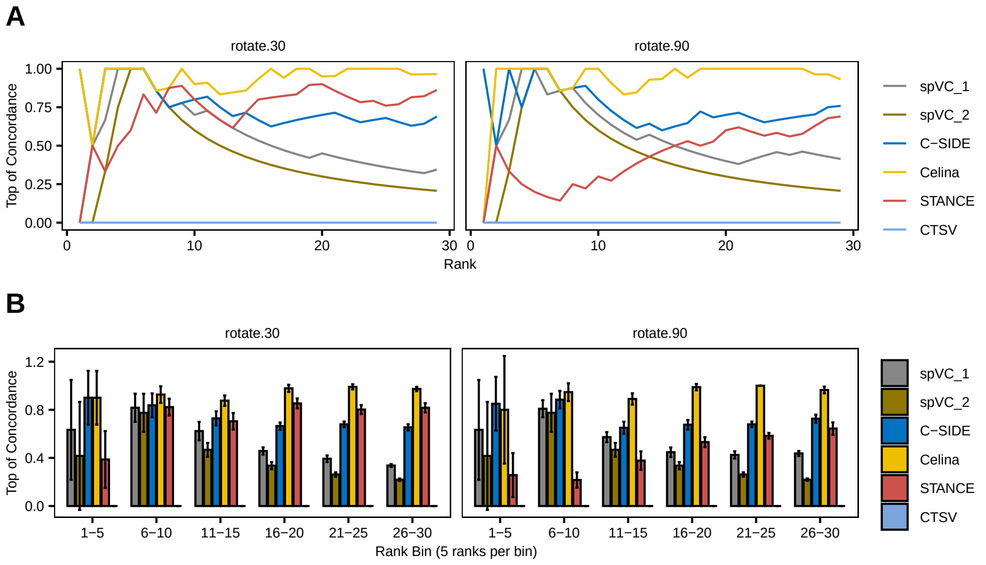

Fig. S16 Concordance at the top between the original and rotated (30° and 90°) Slide-seq tumor dataset, based on the top 30 genes ranked by adjusted p-value.

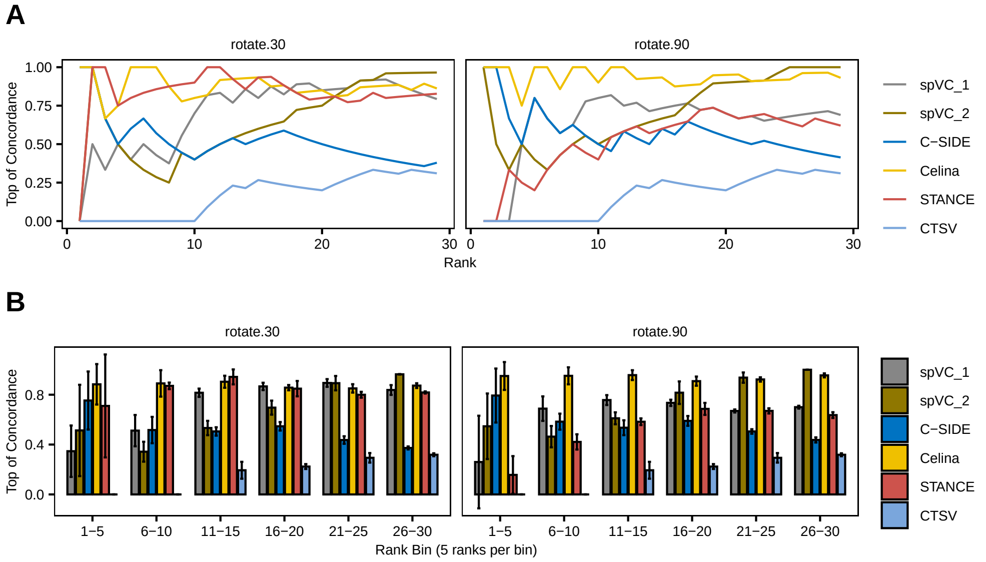

Fig. S17 Concordance at the top between the original and rotated (30° and 90°) Slide-seqV2 hippocampus dataset, based on the top 30 genes ranked by adjusted p-value.

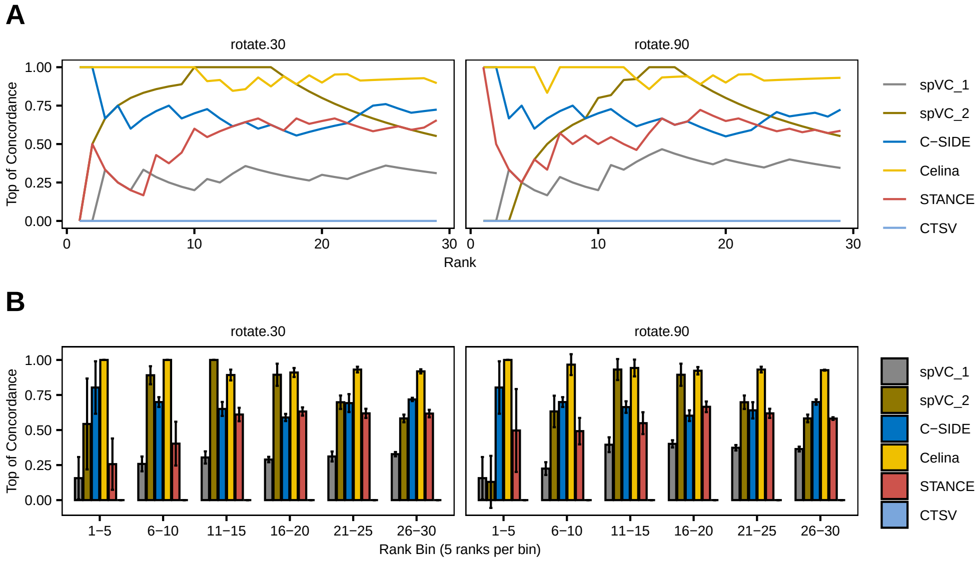

Fig. S18 Concordance at the top between the original and rotated (30° and 90°) Slide-seqV2 mouse olfactory bulb dataset, based on the top 30 genes ranked by adjusted *p*-value.

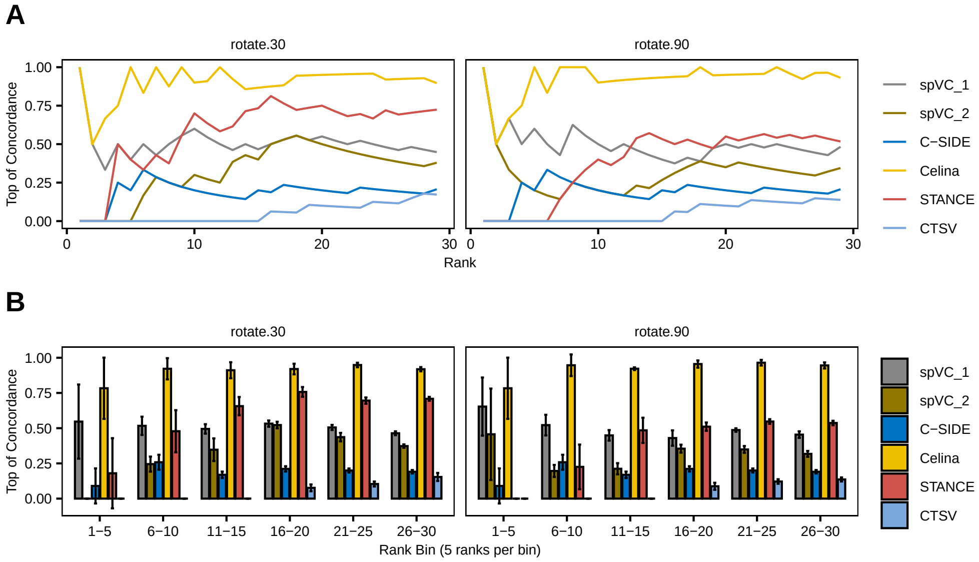

Fig. S19 Concordance at the top between the original and rotated (30° and 90°) spatial transcriptomics developmental heart dataset, based on the top 30 genes ranked by adjusted *p*-value.

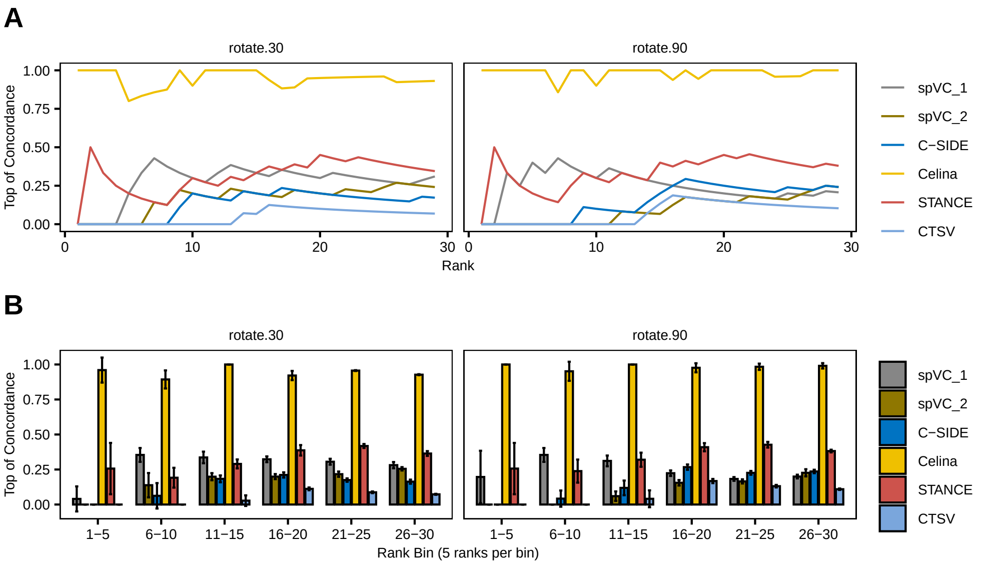

Fig. S20 Concordance at the top between the original and rotated (30° and 90°) Visium mouse brain dataset, based on the top 30 genes ranked by adjusted p-value.

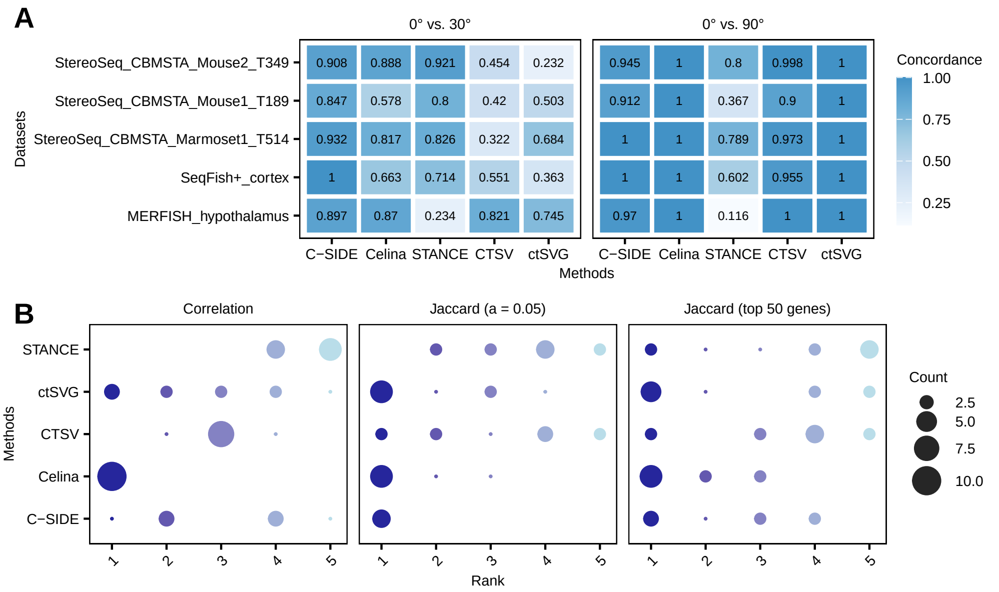

Fig. S21 Heatmap showing the mean CAT scores (top 30 genes) upon two rotations (30° and 90°) across the five cell-level datasets.

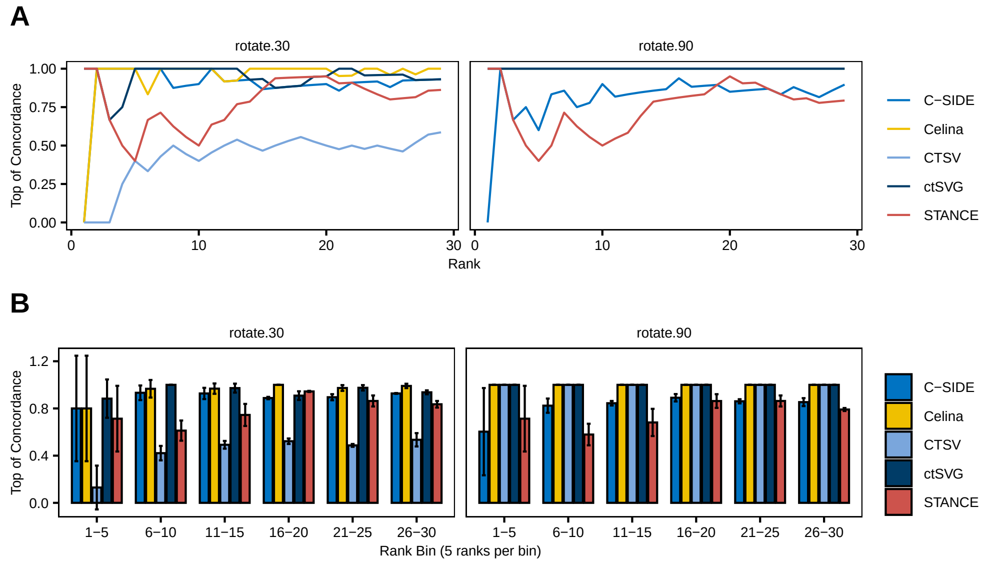

Fig. S22 Concordance at the top between the original and rotated (30° and 90°) MERFISH hypothalamus dataset, based on the top 30 genes ranked by adjusted p-value.

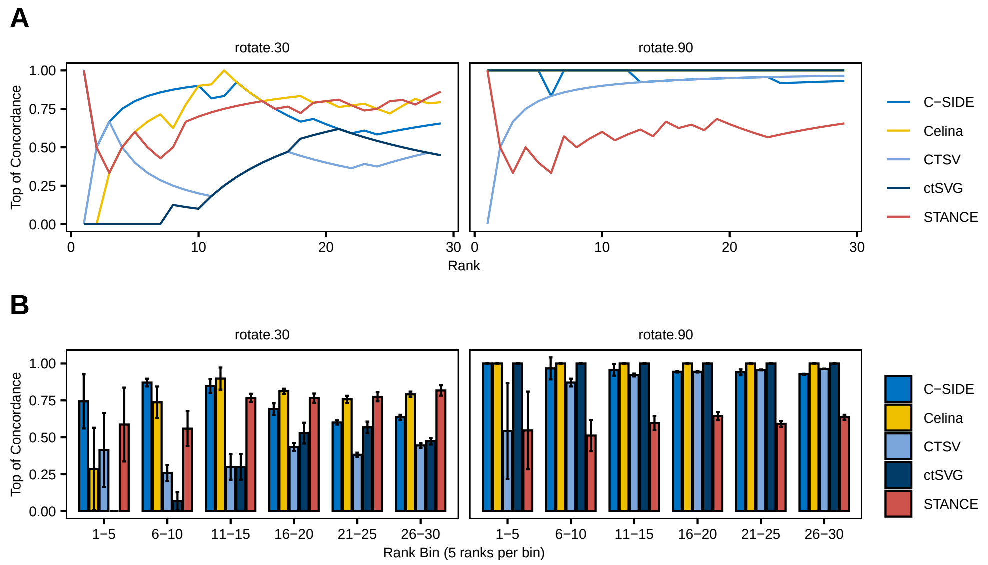

Fig. S23 Concordance at the top between the original and rotated (30° and 90°) SeqFISH+ cortex dataset, based on the top 30 genes ranked by adjusted p-value.

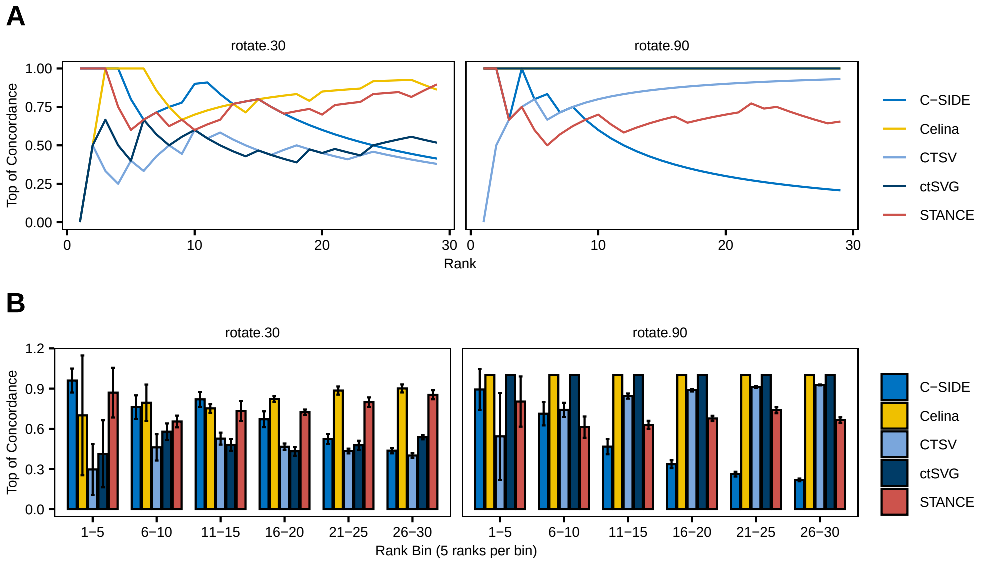

Fig. S24 Concordance at the top between the original and rotated (30° and 90°) Stereo-seq CBMSTA marmoset dataset (sample T514), based on the top 30 genes ranked by adjusted *p*-value.

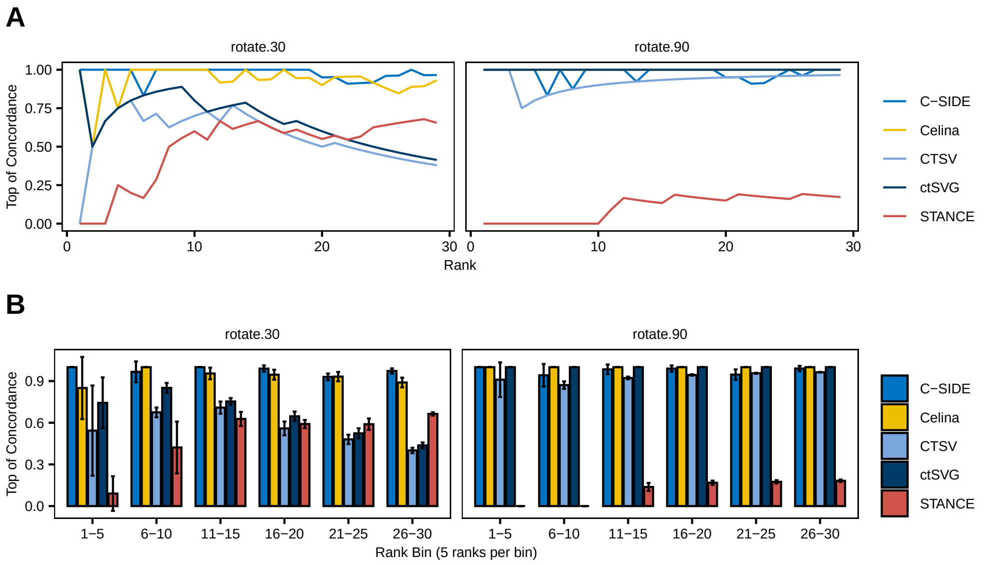

Fig. S25 Concordance at the top between the original and rotated (30° and 90°) Stereo-seq CBMSTA mouse dataset (sample T189), based on the top 30 genes ranked by adjusted *p*-value.

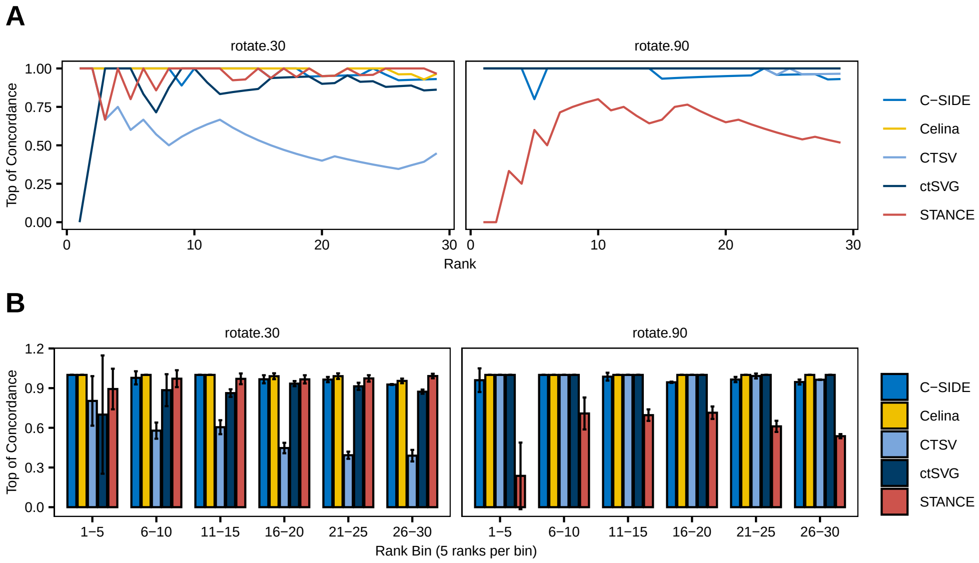

Fig. S26 Concordance at the top between the original and rotated (30° and 90°) Stereo-seq CBMSTA mouse dataset (sample T349), based on the top 30 genes ranked by adjusted p-value.

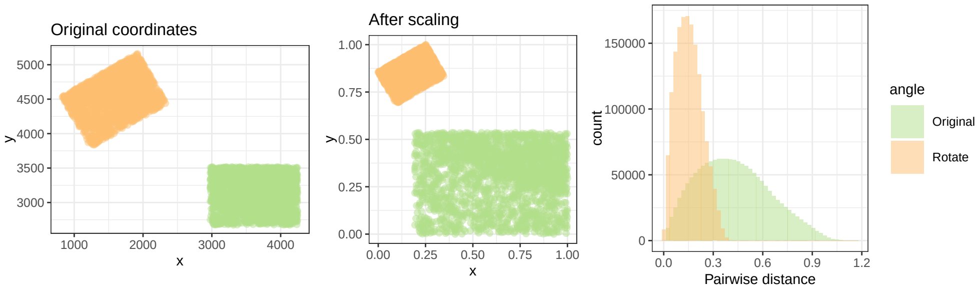

Fig. S27 Left: Scatter plot showing the spatial coordinates of spots before and after rotation. Green and orange points represent the coordinates before and after rotation, respectively. Rotation does not change the Euclidean distance between any two spots. Middle: Scatter plot showing the scaled spatial coordinates of spots before and after rotation. When the x and y ranges differ between before and after rotation, because STANCE uses different scaling factors in this case, the Euclidean distances between spots are no longer preserved. Right: Distribution of pairwise distances between spots before and after rotation.

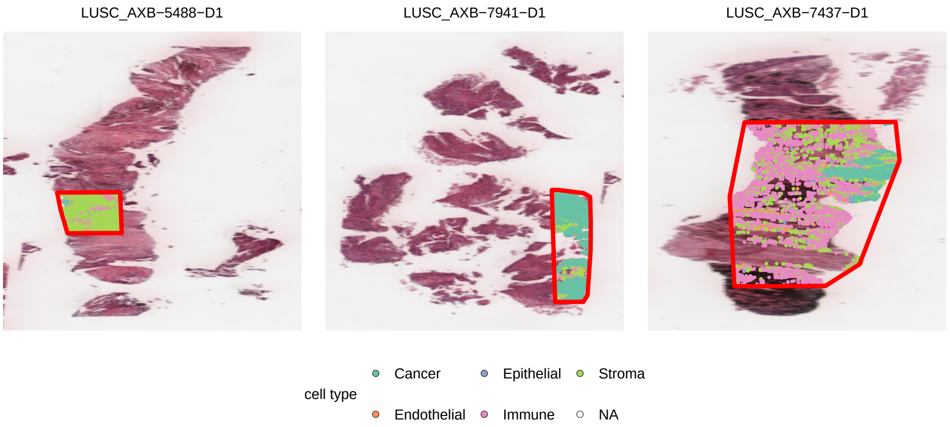

Fig. S28 Tissue images of three LUSC slides. The regions highlighted with red boxes indicate the areas used for deconvolution and ctSVG analyses, where dots in different colors represent distinct cell types.

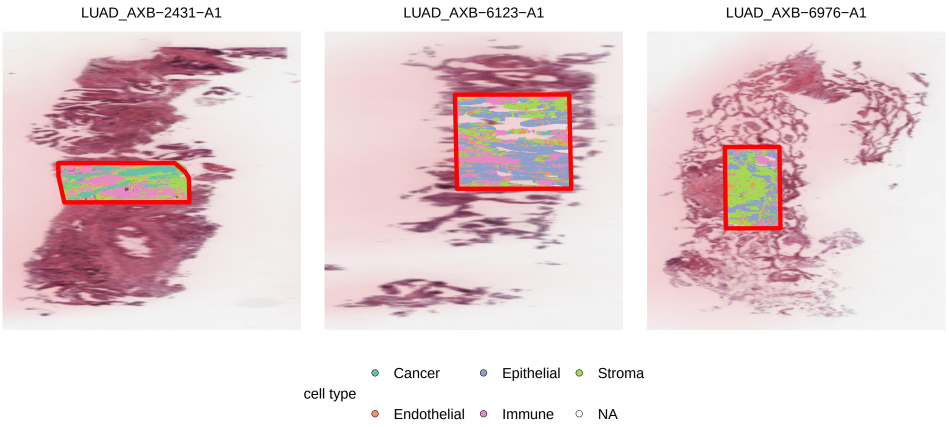

Fig. S29 Tissue images of three LUAD slides. The regions highlighted with red boxes indicate the areas used for deconvolution and ctSVG analyses, where dots in different colors represent distinct cell types.

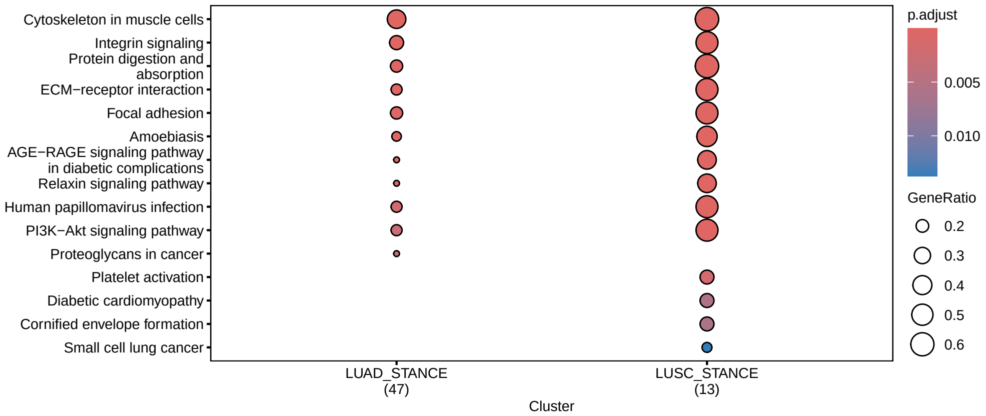

Fig. S30 Dot plots showing the KEGG functional enrichment of conserved genes identified by STANCE in LUAD and LUSC, respectively. The size of each dot represents the number of genes, while the color intensity indicates the significance of the p-values, with darker colors corresponding to higher statistical significance.

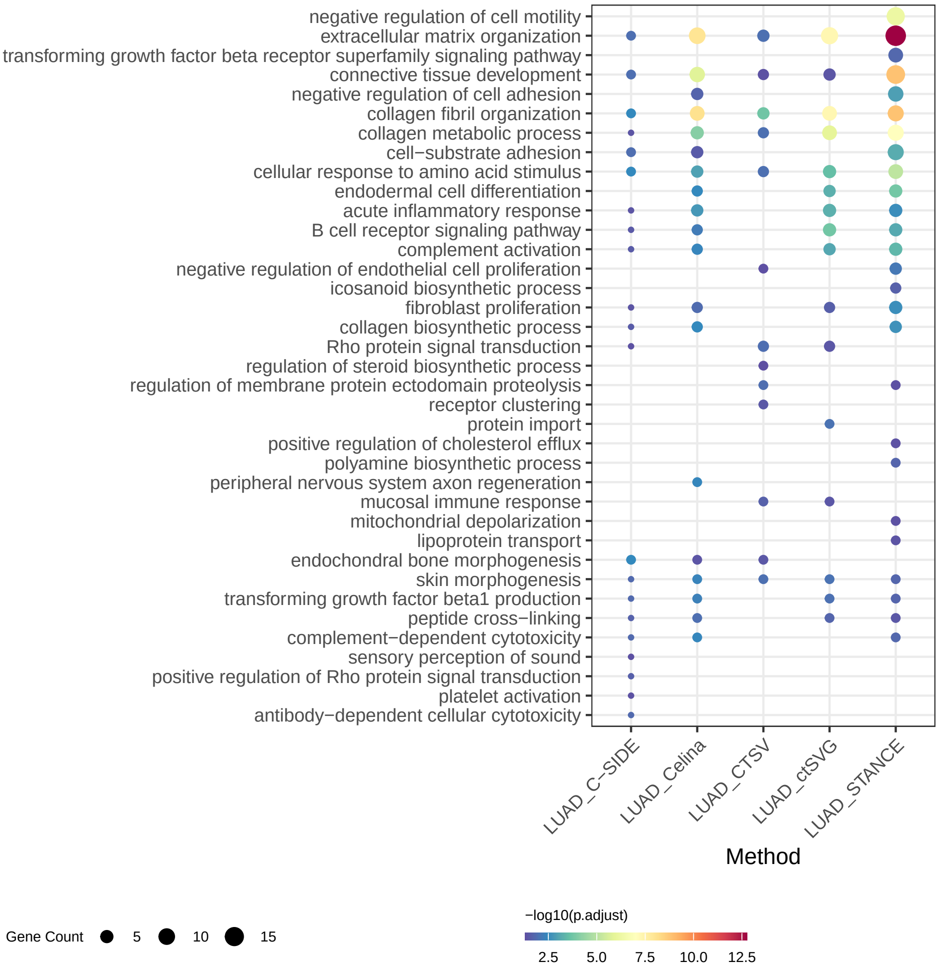

Fig. S31 Dot plots showing enriched GO biological process (BP) of conserved genes identified by each method in three LUAD patients. spVC did not identify any conserved genes. Only pathways enriched in at least one method are shown. Dot color indicates pathway significance, and dot size represents the number of enriched genes.

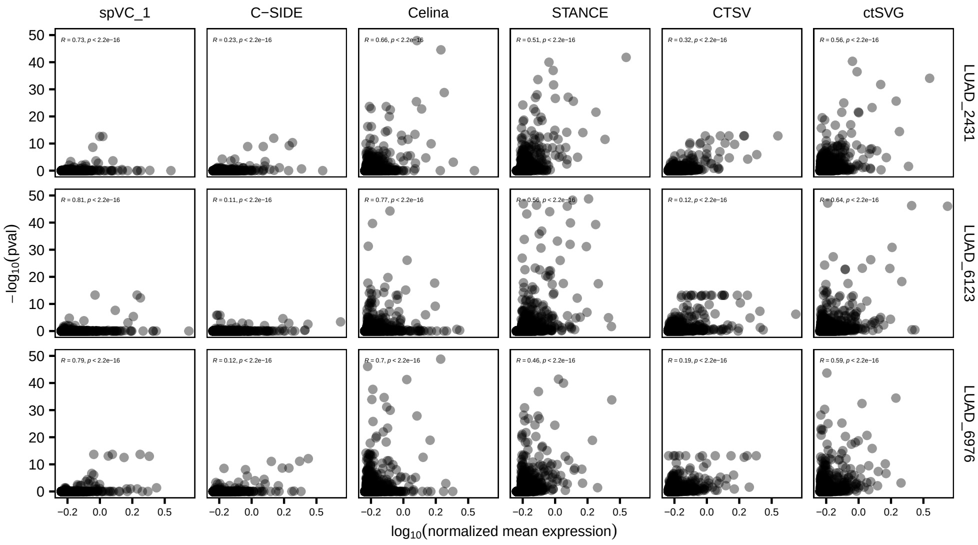

Fig. S32 Scatter plots showing the Spearman's correlation between p-values and expression levels in the three LUAD sections, grouped by each method.

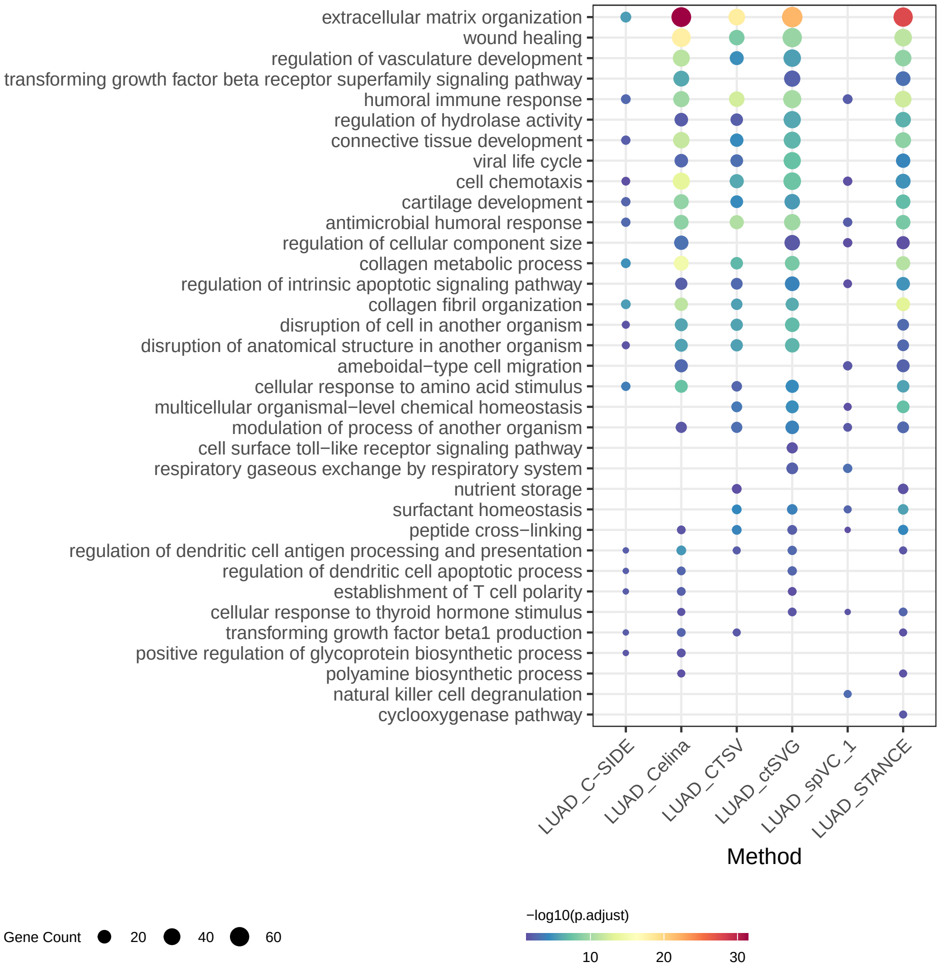

Fig. S33 Dot plots showing enriched GO biological process (BP) pathways of significant ctSVGs identified by each method in lung cancer. Only GO BP pathways enriched in at least one method are shown. Dot color indicates pathway significance, and dot size represents the number of enriched genes.

References

[1] Biermann, J. *et al.* Dissecting the treatment-naive ecosystem of human melanoma brain metastasis. *Cell* **185**, 2591–2608.e30 (2022).

[2] Cable, D. M. *et al.* Cell type-specific inference of differential expression in spatial transcriptomics. *Nature Methods* **19**, 1076–1087 (2022).

[3] Wang, I.-H. *et al.* Spatial transcriptomic reconstruction of the mouse olfactory glomerular map suggests principles of odor processing. *Nature Neuroscience* **25**, 484–492 (2022).

[4] Ji, A. L. *et al.* Multimodal Analysis of Composition and Spatial Architecture in Human Squamous Cell Carcinoma. *Cell* **182**, 497–514.e22 (2020).

[5] Topchyan, P. *et al.* Spatial transcriptomics demonstrates the role of CD4 T cells in effector CD8 T cell differentiation during chronic viral infection. *Cell Reports* **41**, 111736 (2022).

[6] Tabula Muris Consortium *et al.* Single-cell transcriptomics of 20 mouse organs creates a Tabula Muris. *Nature* **562**, 367–372 (2018).

[7] Gouin, K. H. *et al.* An N-Cadherin 2 expressing epithelial cell subpopulation predicts response to surgery, chemotherapy and immunotherapy in bladder cancer. *Nature Communications* **12**, 4906 (2021).

[8] Hou, X. *et al.* Integrating Spatial Transcriptomics and Single-Cell RNA-seq Reveals the Gene Expression Profling of the Human Embryonic Liver. *Frontiers in Cell and Developmental Biology* **9** (2021).

[9] Popescu, D.-M. *et al.* Decoding human fetal liver haematopoiesis. *Nature* **574**, 365–371 (2019).

[10] Lee, H.-O. *et al.* Lineage-dependent gene expression programs influence the immune landscape of colorectal cancer. *Nature Genetics* **52**, 594–603 (2020).

[11] 10x Genomics. Visium human colorectal cancer (2020). Available at: https://www.10xgenomics.com/datasets/human-colorectal-cancer-whole-transcriptome-analysis-1-standard-1-2-0 (Accessed: 2020-10-27).

[12] Sindelka, R. *et al.* Characterization of regeneration initiating cells during Xenopus laevis tail regeneration. *Genome Biology* **25**, 251 (2024).

[13] Ng, M. S. F. *et al.* Deterministic reprogramming of neutrophils within tumors. *Science (New York, N.Y.)* **383**, eadf6493 (2024).

[14] Moffitt, J. R. *et al.* Molecular, spatial, and functional single-cell profiling of the hypothalamic preoptic region. *Science* **362**, eaau5324 (2018).

[15] Eng, C.-H. L. *et al.* Transcriptome-scale super-resolved imaging in tissues by RNA seqFISH+. *Nature* **568**, 235–239 (2019).

[16] Moncada, R. *et al.* Integrating microarray-based spatial transcriptomics and single-cell RNA-seq reveals tissue architecture in pancreatic ductal adenocarcinomas. *Nature Biotechnology* **38**, 333–342 (2020).

[17] Asp, M. *et al.* A Spatiotemporal Organ-Wide Gene Expression and Cell Atlas of the Developing Human Heart. *Cell* **179**, 1647–1660.e19 (2019).

[18] 10x Genomics. Human lymph node (2020). Available at: https://www.10xgenomics.com/datasets/human-lymph-node-1-standard-1-1-0 (Accessed: 2020-06-23).

[19] 10x Genomics. Mouse brain serial section 1 (sagittal-anterior) (2020). Available at: https://www.10xgenomics.com/datasets/mouse-brain-serial-section-1-sagittal-anterior-1-standard-1-1-0.

[20] Hunter, M. V., Moncada, R., Weiss, J. M., Yanai, I. & White, R. M. Spatially resolved transcriptomics reveals the architecture of the tumor-microenvironment interface. *Nature Communications* **12**, 6278 (2021).

[21] Zhao, T. *et al.* Spatial genomics enables multi-modal study of clonal heterogeneity in tissues. *Nature* **601**, 85–91 (2022).

[22] Tasic, B. *et al.* Adult mouse cortical cell taxonomy revealed by single cell transcriptomics. *Nature Neuroscience* **19**, 335–346 (2016).

[23] Chen, A. *et al.* Spatiotemporal transcriptomic atlas of mouse organogenesis using DNA nanoball-patterned arrays. *Cell* **185**, 1777–1792.e21 (2022).

[24] Wang, Y. *et al.* A spatial transcriptome map of the developing maize ear. *Nature Plants* **10**, 815–827 (2024).

[25] Hao, S. *et al.* Cross-species single-cell spatial transcriptomic atlases of the cerebellar cortex. *Science* **385**, eado3927 (2024).
